# Development and implementation of optimized endogenous contrast sequences for delineation in adaptive radiotherapy on a 1.5T MR-linear-accelerator: a prospective R-IDEAL stage 0-2a quantitative/qualitative evaluation of *in vivo* site-specific quality-assurance using a 3D T2 fat-suppressed platform for head and neck cancer

**DOI:** 10.1117/1.JMI.10.6.065501

**Published:** 2023-11-06

**Authors:** Joint Head and Neck Radiotherapy-MRI Development Cooperative, Travis C. Salzillo, M. Alex Dresner, Ashley Way, Kareem A. Wahid, Brigid A. McDonald, Sam Mulder, Mohamed A. Naser, Renjie He, Yao Ding, Alison Yoder, Sara Ahmed, Kelsey L. Corrigan, Gohar S. Manzar, Lauren Andring, Chelsea Pinnix, R. Jason Stafford, Abdallah S. R. Mohamed, John Christodouleas, Jihong Wang, Clifton David Fuller

**Affiliations:** a MD Anderson Cancer Center, Radiation Oncology, Houston, Texas, United States; b Philips Healthcare, Cleveland, Ohio, United States; c MD Anderson Cancer Center, Radiation Physics, Houston, Texas, United States; d MD Anderson Cancer Center, Imaging Physics, Houston, Texas, United States; e Elekta AB, Stockholm, Sweden

**Keywords:** magnetic resonance imaging/linear accelerator, fat suppression, head and neck cancer

## Abstract

**Purpose:**

To improve segmentation accuracy in head and neck cancer (HNC) radiotherapy treatment planning for the 1.5T hybrid magnetic resonance imaging/linear accelerator (MR-Linac), three-dimensional (3D), T2-weighted, fat-suppressed magnetic resonance imaging sequences were developed and optimized.

**Approach:**

After initial testing, spectral attenuated inversion recovery (SPAIR) was chosen as the fat suppression technique. Five candidate SPAIR sequences and a nonsuppressed, T2-weighted sequence were acquired for five HNC patients using a 1.5T MR-Linac. MR physicists identified persistent artifacts in two of the SPAIR sequences, so the remaining three SPAIR sequences were further analyzed. The gross primary tumor volume, metastatic lymph nodes, parotid glands, and pterygoid muscles were delineated using five segmentors. A robust image quality analysis platform was developed to objectively score the SPAIR sequences on the basis of qualitative and quantitative metrics.

**Results:**

Sequences were analyzed for the signal-to-noise ratio and the contrast-to-noise ratio and compared with fat and muscle, conspicuity, pairwise distance metrics, and segmentor assessments. In this analysis, the nonsuppressed sequence was inferior to each of the SPAIR sequences for the primary tumor, lymph nodes, and parotid glands, but it was superior for the pterygoid muscles. The SPAIR sequence that received the highest combined score among the analysis categories was recommended to Unity MR-Linac users for HNC radiotherapy treatment planning.

**Conclusions:**

Our study led to two developments: an optimized, 3D, T2-weighted, fat-suppressed sequence that can be disseminated to Unity MR-Linac users and a robust image quality analysis pathway that can be used to objectively score SPAIR sequences and can be customized and generalized to any image quality optimization protocol. Improved segmentation accuracy with the proposed SPAIR sequence will potentially lead to improved treatment outcomes and reduced toxicity for patients by maximizing the target coverage and minimizing the radiation exposure of organs at risk.

## 1 Introduction

Radiotherapy treatment planning using magnetic resonance imaging (MRI), alone or in combination with computed tomography (CT), has become increasingly common over the past couple of decades.^1^^–^^4^ The superior soft tissue contrast of MRI compared with CT makes it an attractive imaging modality for target structure and organ-at-risk (OAR) segmentation.^5^^–^^7^ Furthermore, recent advances in deformable image registration to spatially accurate CT images and electron density assignment using synthetic CT generation or alternative atlas-based approaches have helped address the primary pitfalls of combined magnetic resonance (MR)/CT-based treatment planning, namely geometric distortion and direct dose estimation.^8^^–^^16^

MR-linear accelerator (Linac) users are major beneficiaries of these advances in MR-based treatment planning.^17^^,^^18^ These hybrid MRI-Linac devices can acquire imaging data during each fraction of radiotherapy and incorporate these data into MR-compatible treatment planning systems.^19^^,^^20^ Moreover, these daily images can be used in online or offline adaptive planning workflows when major changes in anatomy or tumor function are detected.^21^^,^^22^ Thus a major area of research focuses on sequence development for MR-Linac devices to improve the visualization of relevant structures and discover and acquire useful imaging biomarkers for treatment response and resistance.^23^^,^^24^

The treatment of head and neck cancer (HNC), especially human papillomavirus-associated HNC, using MR-Linac has been particularly successful.^25^^–^^27^ These tumors are relatively radiosensitive, which warrants the use of the adaptive replanning utility with the MR-Linac.^28^^–^^30^ Furthermore, delineation of the complex anatomy in the head and neck region is difficult to visualize with CT, with which most of the structures have a uniform signal and little contrast. Conversely, T2-weighted (T2w) MRI provides a higher signal-to-noise ratio as well as contrast with surrounding structures, which allows for clearer and more precise segmentation.^31^^,^^32^ However, fat also appears hyperintense on these images, which can reduce the contrast in structures adjacent to fat and obfuscate their boundaries.^33^^,^^34^ Fat is present in many areas in the head and neck such as at the skull base and fat pads in the face, the parapharyngeal space, the retropharyngeal space, and the submucosal spaces of the supraglottic larynx.^35^ Thus for accurate segmentation of the target and OAR during radiotherapy planning, the use of fat-suppressed images is necessary.

To attenuate the fat signal while keeping the water signal within tissue intact, several fat-suppression methods have been established. These methods, which use prepulse inversion recovery and/or bandwidth strategies during image acquisition or postprocessing techniques during image reconstruction, have been described at length in the literature.^36^^–^^41^ These include the short tau inversion recovery (STIR), chemically selective saturation (CHESS), spectral presaturation with inversion recovery (SPIR), spectral attenuated inversion recovery (SPAIR), and Dixon techniques. STIR suffers from a lower signal-to-noise ratio (SNR) because of the attenuation of all tissue signals with the same T1 as fat, but the technique is less sensitive to $B_{0}$ inhomogeneities. CHESS improves the SNR by selectively attenuating the fat signal, but it suffers substantial effects of $B_{0}$ and $B_{1}$ inhomogeneities. SPIR is a hybrid of STIR and CHESS but suffers from the same $B_{0}$ and $B_{1}$ inhomogeneities as CHESS. Depending on the severity of the artifact, the strength of the fat suppression can be adjusted. SPAIR, like SPIR, selectively attenuates the fat signal, but it does so using an adiabatic pulse, which helps offset the effects of $B_{1}$ inhomogeneities at the expense of a higher specific absorption rate (SAR) in the patient. SAR levels are typically capped according to level 1 and 2 power settings on the console. The Dixon technique results in moderate SNR, more robust fat suppression, and less sensitivity to both $B_{0}$ and $B_{1}$ inhomogeneities (with the help of postprocessing), although it usually requires longer scan times due to the acquisition of multiple images.

Although fat-suppressed sequences are commonly used in diagnostic systems, there has yet to be a sequence specific for head and neck treatment planning on the MR-Linac presented in the literature. Thus this study, whose purpose is to develop and optimize a three-dimensional (3D), fat-suppressed, T2w sequence that could be used on a 1.5 T Unity MR-Linac (Elekta AB, Stockholm, Sweden) for treatment planning purposes, is warranted and clinically impactful. Cast in the R-IDEAL (radiotherapy-predicate studies, idea, development, exploration, assessment, long-term study) framework, as per by the MR-Linac Consortium recommendations, this study is designed to stage 0 (radiotherapy predicate studies) to stage 2a (development).^42^ This study provides a methodological and rigorous foundation for the implementation of this technical development for MR-Linac clinical workflows and a starting point for future studies along the R-IDEAL pipeline, which is an assessment methodology for the evidence-based clinical evaluation of innovations in radiation oncology. The image quality analyses of the fat-suppressed sequence, along with the exam card of the optimized sequence itself, are presented here, so the sequence may be disseminated to Unity MR-Linac users.

A secondary goal of this study is to develop a comprehensive and robust image quality analysis platform to objectively score and rank candidate fat-suppressed sequences. Image quality assessment is generally split into 2 classes: subjective approach (which is based on human perception) and objective approach (based on mathematical concepts).^43^^,^^44^ Subjective approaches more closely represent the human visual system (HVS) and thus are arguably more reliable for grading images. However, there is a degree of variability between observer perceptions, and the overall grading process can be highly time consuming. Objective approaches minimize bias and analysis time, but typically measure individual aspects of image quality, which variably correlate to the HVS. There are efforts to effectively model the HVS with an objective approach,^44^^–^^46^ but optimal solutions have not yet been determined. As such, most sequence development and optimization studies report one or more metrics commonly used by physicists, including SNR, contrast-to-noise ratio (CNR), and modulation transfer function.^47^ The correlation between subjective and objective approaches has also been evaluated.^48^ To consolidate the strengths of both approaches, we include both subjective and objective metrics relevant to radiotherapy treatment planning in our assessment. This includes the metric known as conspicuity, which is a parameter directly related to the visibility of an object in a complex background. Although such a variable would be directly applicable to radiotherapy segmentation, it is not commonly reported, nor is it available on most image analysis platforms. Thus we develop a script to automatically calculate this value from an image and its associated segmentations. This metric, along with SNR, CNR, and pairwise distance, are incorporated to capture multiple aspects of the HVS. By calculating the metrics separately and combining them in a rubric, specific strengths and deficiencies of each sequence can be observed. The resultant analysis platform described here is easily customizable and can be generalized for the optimization of any anatomic-based imaging sequence, adding further value to this study.

## 2 Methods

### 2.1 Data Availability

All patient images and segmentations were anonymized and uploaded to FigShare.

### 2.2 Sequence Development and Optimization

A standard 3D, T2w, turbo spin echo sequence was used as an initial template for the fat-suppressed sequence. A Philips MR console emulation software program (Philips Healthcare, Best, The Netherlands) was used to modify sequence parameters and simulate relative image properties, such as the SNR. The first parameter that was iterated was the fat-suppression method. Because there is no clinically available 3D Dixon sequence for the Unity device, only SPAIR and STIR techniques were investigated because of their relative resistance to the $B_{0}$ and $B_{1}$ inhomogeneities that are known to occur in the head and neck region in MRI. Initial image acquisitions demonstrated that the overall image quality for the SPAIR fat-suppression method was superior to that of the STIR method, especially with regards to SNR, so subsequent sequence optimization was limited to SPAIR sequences. The expertise of an MR physicist was used to logically iterate through several parameters to produce candidate SPAIR iterations, which satisfied the following constraints: 5- to-6-min acquisition time, ∼1  mm isotropic reconstructed resolution, and TE and TR values for T2 weighting. A preliminary round of image acquisition and qualitative analysis eliminated sequences that produced severe artifacts or insufficient image quality. Five SPAIR sequences (SPAIR 1 to 5) were chosen as the final candidate sequences for further analysis. The parameters of these sequences are given in Table 1.

**Table 1:** Relevant pulse sequence parameters for the nonsuppressed, T2-weighted sequence, and candidate SPAIR sequences.

Sequence parameter	Non-FS	SPAIR 1	SPAIR 2	SPAIR 3	SPAIR 4	SPAIR 5
Scan mode	3D	3D				
Technique	TSE	TSE				
Fat suppression	N/A	SPAIR				
In-plane FOV (mm)	520 × 298	520 × 270				
In-plane acq. resolution (mm)	1.2 × 1.2	1.4 × 1.5				
Through-plane FOV (mm)	250	200	200	200	200	300
Through-plane acq. resolution (mm)	2.2	2.0	2.0	2.0	2.0	2.4
Reconstructed voxel size (mm)	0.7 × 0.7 × 1.1	0.8 × 0.8 × 1.0	0.8 × 0.8 × 1.0	1.0 × 1.0 × 1.0	0.8 × 0.8 × 1.0	1.0 × 1.0 × 1.2
Oversample factor	1.3	1.4	1.3	1.3	1.4	1.3
TSE factor	150	72	76	66	72	76
FID reduction	Default	Strong	Through-plane	Strong	Strong	Through-plane
Flip angle (deg)	90	90				
Refocusing angle (deg)	30	40	40	40	55	40
TEeff/TEequiv (ms)	375/143	182/93	190/96	185/95	182/107	190/96
TR (ms)	2100	1600	1600	1400	1400	1400
WFS (pix)/BW (Hz)	0.473/459.3	0.407/533.4	0.456/476.6	0.498/436.4	0.407/533.4	0.459/473.3
NSA	2	2				
Scan duration	6:03	5:52	5:09	5:05	5:09	5:33
SAR (W/kg)	0.166	0.216	0.220	0.239	0.328	0.251

Abbreviations: acq, acquisition; BW, bandwidth; eff, effective; equiv, equivalent; FID, free induction decay; FOV, field of view; N/A, not applicable; NSA, number scan averages; Non-FS, non-suppressed; SPAIR, spectral attenuated inversion recovery; TE, time-to-echo; TR, repetition time; TSE, turbo spin echo; and WFS, water-fat shift.

### 2.3 Image Acquisition

Image data for the preliminary and main analyses were acquired from HNC patients who were enrolled in the MOMENTUM clinical trial (NCT04075305) at our institution and provided informed consent. This study was approved by the MD Anderson Cancer Center Institutional Review Board (Protocols PA15-0418 and PA18-0341). The images were acquired during patients’ MR simulation and daily treatment fractions on the 1.5 T Unity MR-Linac. Patients were immobilized with a customized head and shoulder Klarity AccuCushion (Klarity Medical Products, Newark, Ohio, United States), a thermoplastic head neck and shoulder mask (Orfit Industries America, Wijnegem, Belgium), and a customized bite-block attachment fixated to the thermoplastic mask, which attached to a preheated moldable bite-block (Precise Bite, Civico, Coralville, Iowa, United States) that conformed to the patient’s upper teeth. To avoid keeping patients on the treatment table for an extended period of time, between 1 and 3 nonsuppressed/SPAIR images were acquired in a fraction. One nonsuppressed image and one image from each SPAIR iteration were incorporated into the analysis per patient. The scanner is equipped with four-channel radiolucent radiofrequency coils positioned anteriorly and posteriorly to the patient, which is standard for Unity devices. A nonsuppressed T2w sequence and five SPAIR T2w sequences (SPAIR1 to 5) were acquired for each of five patients with HNC.

### 2.4 MR Physicist Initial Screening

Two MR physicists independently analyzed patient images from the five SPAIR sequences according to a rubric (Appendix 2 in the Supplementary Material) that asked the physicists to qualitatively rank each sequence, according to their preference. Additionally, the physicists were asked to identify any artifacts that were present in the image. One physicist quantified the number of slices that were affected by burnout (loss of the tissue signal due to improper fat suppression) anteriorly and posterolaterally. The purpose of this screening was to identify persistent image quality deficiencies, including severe artifacts, that would disqualify the associated sequences from further analysis.

### 2.5 Image Segmentation

For the sequences that did not possess substantial image quality deficiencies, five postgraduate physicians in radiation oncology used Raystation software (Raysearch Laboratories AB, Stockholm, Sweden) to segment the gross primary tumor volume (GTV), metastatic lymph nodes, left and right parotid glands, and left and right pterygoid muscles, which are relevant to radiotherapy treatment planning. The physicians (referred to hereafter as segmentors) were restricted from looking at each other’s segmentations but were allowed to refer to a radiologist’s report for structure identification (which is a common clinical occurrence). Furthermore, the segmentors were asked to recontour each structure segmentation from scratch on each image rather than propagating the segmentations onto each image and modifying them. A segmented structure on a particular sequence is referred hereafter as a sequence-structure pair. A nonresident researcher also segmented an air-filled cavity within the trachea using 10 slices for each image. These segmentations were used for noise calculations because the areas surrounding the patient were automatically masked in postprocessing before image export. Additionally, the nonresident researcher segmented three areas of cheek and neck fat on 1 slice per patient for CNR measurements. The noise and fat segmentations are illustrated in Fig. S1 in the Supplementary Material. These segmentations were reviewed for accuracy by an experienced physician with more than 10 years of experience in HNC radiation oncology.

### 2.6 Quantitative Image Quality Analyses

#### 2.6.1 SNR and CNR measurements

The SNR of each structure was calculated as the mean signal of the sequence-structure pair divided by the standard deviation of the noise segmentation (10 slices of the air-filled cavity in the trachea). The SNR was also calculated for the fat segmentations for each sequence as a measure of fat suppression. The CNR measurements between each structure and both fat and muscle were also determined by calculating the SNR difference between the structure and fat or muscle. The muscle segmentation for these calculations was the mean signal of the pterygoid muscle sequence-structure pair. Because these metrics are segmentor-agnostic, the voxels within each segmentor’s region-of-interest (ROI) were averaged together for a given sequence-structure pair for mean signal calculations and pooled for each patient to be used in the statistical analysis. Thus the sample size for this calculation was equivalent to the number of patients imaged.

#### 2.6.2 Conspicuity measurements

Conspicuity is a measurement of the ratio between the ROI contrast and the surrounding signal complexity. It is thought to be a more robust descriptor of structure visibility than the SNR and CNR. A script to calculate conspicuity was developed according to the equations first described by Revesz et al.^49^ and is available at https://github.com/tcsalzillo/ConspicuityAnalysis. The original structure segmentations were isotropically expanded and contracted by 1 and 2 mm using Velocity AI software (Varian Medical Systems, Inc., Palo Alto, California, United States). Because conspicuity was first formulated for two-dimensional images, the conspicuity for each slice occupied by the structure for a particular sequence (the sequence-structure pair) was recorded. This stack of conspicuity values for each sequence-structure pair was then pooled for each segmentor and for each patient, filtered to the inner 90th percentile (to account for outliers), and inputted in the statistical analysis. Thus the sample size for this calculation was equivalent to 90% × slices per patient × number of patients × number of segmentors.

#### 2.6.3 Pairwise distance metrics

The Dice similarity coefficient (DSC) and 95% Hausdorff distance (HD) metrics for each sequence-structure pair between each segmentor were calculated as previously described.^50^ DSC is defined as

$$\mathrm{DSC}=\frac{2|A\cap B|}{\left|A|+|B\right|},$$

where |A| and |B| are the number of voxels from contoured volumes A and B, respectively, and |A∩B| denotes the number of voxels included in the intersection between volumes A and B. The DSC ranges in values from 0 (no overlap) to 1 (perfect overlap). HD is defined as

$$\mathrm{HD}=\text{percentile}(d_{A,B}\cup d_{B,A},95^{\mathrm{th}}),$$

where $d_{A,B}$ is the vector containing all minimum Euclidian distances from the surface point from volume A to B. HD values closer to zero represent a better agreement between two contours’ surfaces. These metrics are among the most ubiquitous volumetric and surface distance metrics reported in the literature.^51^ The stacks of pairwise DSC and HD metrics for each sequence-structure pair were pooled for each patient, filtered to the inner 90th percentile, and inputted in the statistical analysis. Thus the sample size for these calculations was equivalent to 90% × number patients × (number of segmentors choose 2).

### 2.7 Qualitative Image Quality Analyses

#### 2.7.1 Segmentor grading and comments

As each segmentor worked to delineate the structures, they were asked to complete a rubric (Appendix 1 in the Supplementary Material) that asked the segmentor to qualitatively rank each sequence-structure pair, according to their preference, for each patient. Additionally, the segmentor was asked to provide specific comments about the appearance or visibility of a structure. These comments were classified into positive (e.g., “structure X looked great”), neutral (e.g., “structure X looked acceptable”), or negative (e.g., “could not see structure X”) categories. A metric was created to compare the relative amount of positive and negative comments across segmentors for a specific sequence-structure pair, which was calculated with the following equation:

$$\text{segmentor comment metric}=\frac{\#\text{positive comments}-\#\text{negative comments}}{\#\text{possible comments}}\in \{-1,1\}.$$

This formula was derived as a way to reflect the overall qualitative opinion of the sequences while normalizing to the possible number of comments submitted about the sequence. In this study, 4 of the 5 segmentors provided comments, so the denominator was 4. We assumed that all sequences had a baseline neutral opinion, which is why neutral comments were omitted from the numerator of the calculation. This metric can be thought of as a percent favored/unfavored opinion of the sequences. Segmentor grade scores for each sequence-structure pair were pooled by segmentor and by patient and inputted in the statistical analysis. Thus the sample size for this metric was equivalent to number of segmentors × number of patients. The segmentor comment metric incorporated the number of segmentors in the calculation, so the sample size was equivalent to the number of patients.

### 2.8 Statistical Analysis

Each metric was subjected to further statistical analysis, which was performed using GraphPad Prism 8 (GraphPad Software, La Jolla, California, United States). First, the distribution normality was assessed using the Kolmogorov–Smirnov test. If the distributions were normal, the mean value and standard deviation of the metric were calculated. The statistical significance between each sequence-structure pair was then determined using the parametric one-way analysis of variance test with follow-up Tukey multiple comparison corrections. If the distributions were found to be nonnormal, the median value and interquartile range of the metric were calculated. The statistical significance between each sequence-structure pair was determined using the nonparametric Kruskal–Wallis test followed by Dunn multiple comparison corrections. This test was also used to analyze the qualitative segmentor grading and comments analysis. For all distributions, statistical significance was attributed to comparisons that produced a P value <0.05.

### 2.9 Rubric for Overall Sequence Scoring

For each metric that was analyzed, a score was determined for each sequence-structure pair. Each sequence-structure pair received a score between 1 and 4, where 4 corresponded to the pair with the best performance. The value of 4 is equivalent to the number of sequences analyzed. Sequence-structure pairs could only receive a higher score than other pairs if the difference in performance was statistically significant (P<0.05) compared with all other sequence-structure pairs with a lower score. Sequence-structure pairs that received the same score were rescaled to the average rank between them. For example, if a scoring distribution was scored as {4, 2, 2, 1}, it was rescaled to {4, 2.5, 2.5, 1}, where 2.5=(3+2)/2. For clarity, these rescaled scores will be regarded as metric scores.

The metric scores within each analysis category (SNR, CNR, conspicuity, etc.) were summed for each sequence-structure pair. For structure-agnostic metrics, the score was added to each structure within the sequence. The summed metric scores for a particular structure within a sequence was then renormalized to a score between 1 and 4 (4 corresponding to highest summed score) according to its rank relative to the same structure among the other sequences. For clarity, these will be regarded as normalized category scores. This normalization was performed so that a category with more metrics (such as SNR and CNR measurements) would be weighed the same in the overall analysis as a category with fewer metrics (such as conspicuity).

**Fig. 1:**
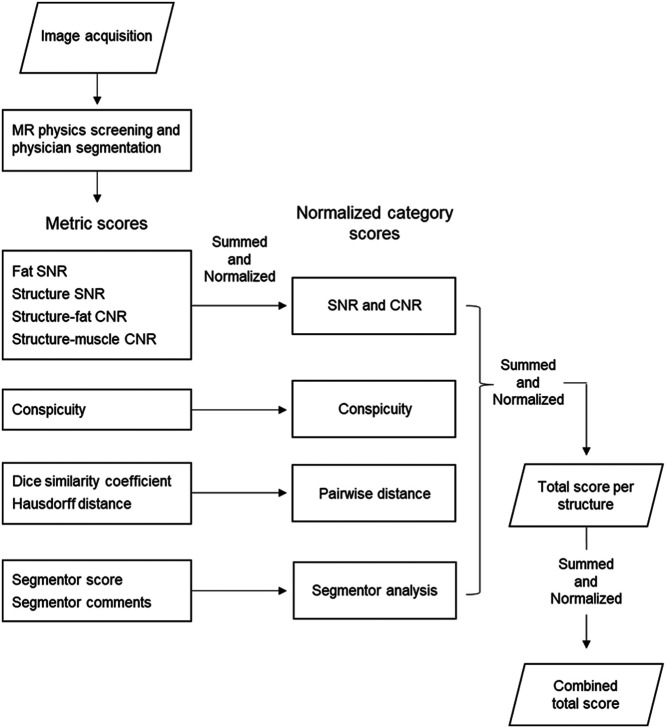
Flowchart of scoring procedure for the analysis platform. Images are acquired and screened by MR physicists. Images from acceptable sequences are segmented by physicians. Metrics across multiple categories are calculated for segmented structures. Metric scores for each sequence-structure pair from each category are summed and normalized to their respective normalized category scores. Normalized category scores are further summed across structures and normalized to calculate the total score for each sequence-structure pair. Finally, the total score per structure within each sequence are summed and normalized to calculate the final combined total score. During each “summed and normalized” step, weights can be applied if the user wishes to weigh an individual metric, individual analysis category, or individual structure higher for their specific application. CNR, contrast-to-noise ratio; MR, magnetic resonance; SNR, signal-to-noise ratio.

The normalized category scores for each sequence-structure pair were then summed and normalized to determine the total score and the normalized total score. These scores were used to compare the overall image quality for each structure among the sequences. Finally, the total score for each structure within a sequence was summed across structures and normalized to determine the combined total score and combined normalized score. These scores were used to compare the overall image quality across structures among the sequences. Refer to Fig. 1 for a graphical depiction of the scoring.

## 3 Results

### 3.1 Image Acquisition and MR Physicist Initial Screening

**Fig. 2:**
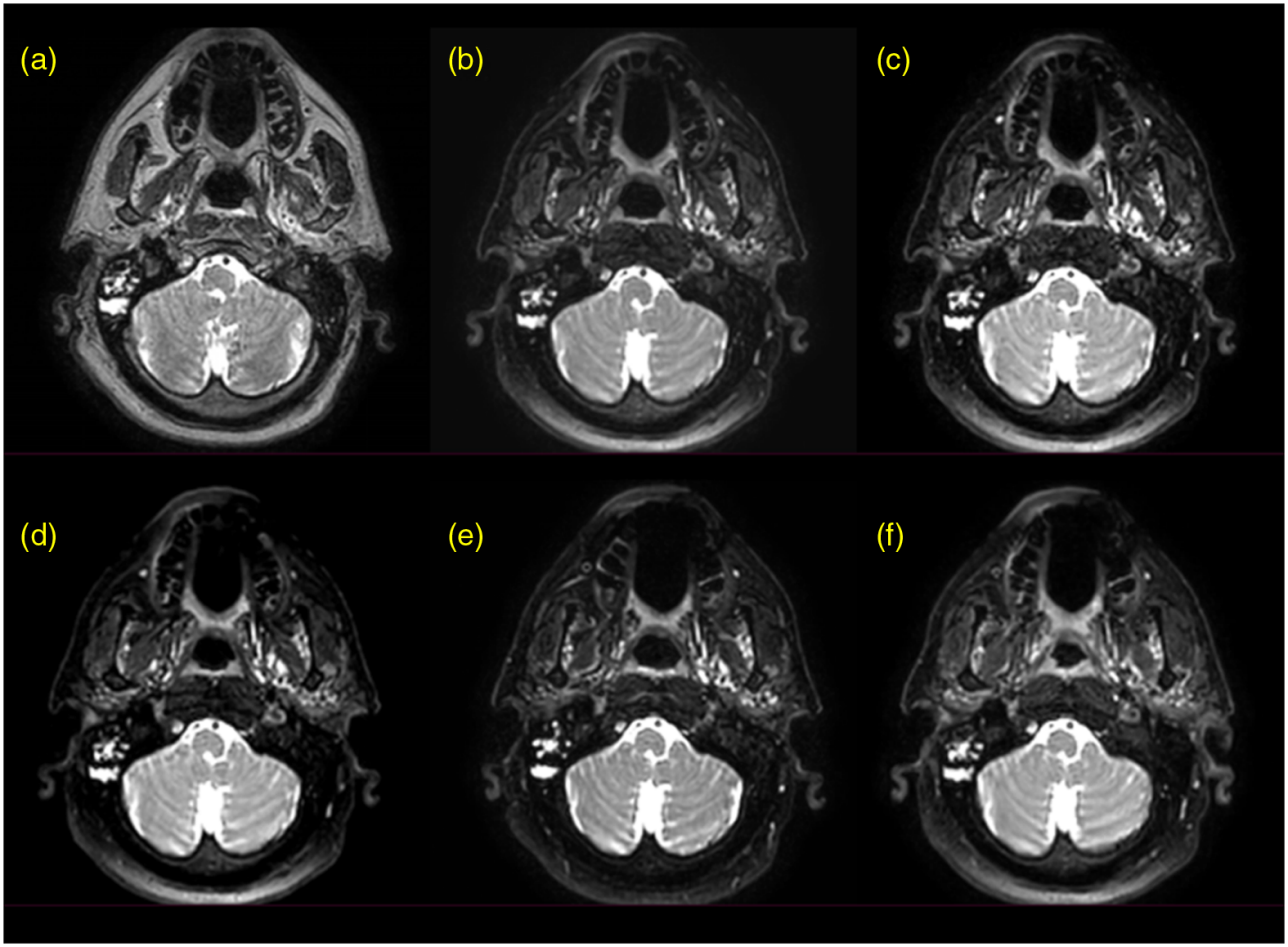
(a) Nonsuppressed, T2-weighted sequence and (b)–(f) five SPAIR sequences acquired on a representative patient with HNC using a Unity magnetic resonance linear accelerator.

**Fig. 3:**
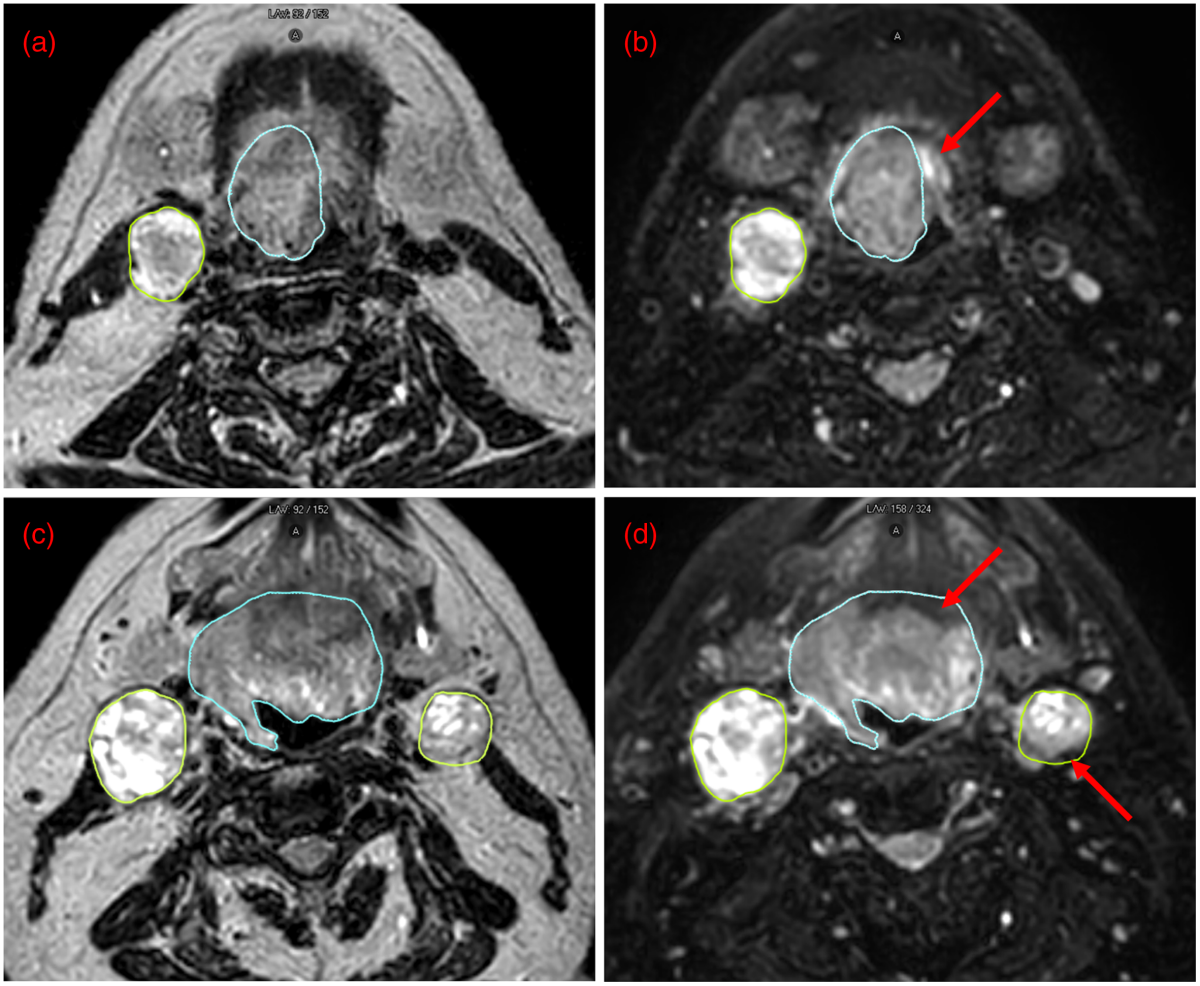
Representative (a), (c) nonsuppressed, T2-weighted and (b), (d) SPAIR T2-weighted images in a patient with HNC. These images are from the SPAIR 4 sequence iterations. The top and bottom rows show two different slices from the patient and illustrate the differences in the clarity of the primary tumor (segmented in blue) and the metastatic lymph nodes (segmented in yellow). The visible segmentations were initially drawn on the nonsuppressed, T2-weighted image. (a), (b) The original segmentation underestimated the extent of the primary tumor, which is clearly visible on the SPAIR image (red arrow in b). The clarity of the inferior portions of the submandibular glands (bilateral structures anterior to the metastatic lymph node) can also be appreciated between the nonsuppressed and SPAIR 4 images. In the nonsuppressed image, the submandibular glands are hypointense with little contrast to surrounding fat, but in the SPAIR 4 image, they are hyperintense with exquisite contrast to the suppressed fat signal. (c), (d) The original segmentations overestimated the extent of the primary tumor and the metastatic lymph node, the boundaries of which are better visualized on the SPAIR 4 images (red arrows in d). Refer to Fig. S1 in the Supplementary Material to see the images without the segmentations visible.

A nonsuppressed T2w sequence and five candidate SPAIR sequences were successfully acquired for each of the five patients. The six sequences for a representative patient are shown in Fig. 2. Paired nonsuppressed and paired SPAIR images (SPAIR 4) in two regions of the head and neck are illustrated in Fig. 3 (with visible segmentations) and Fig. S2 in the Supplementary Material (without visible segmentations). The borders of the primary tumor and metastatic lymph nodes are clearer on the SPAIR image than on the nonsuppressed image. As a result, the segmentations initially drawn on the nonsuppressed image clearly overestimate and/or underestimate the extent of the primary tumor and metastatic lymph nodes when viewed on the SPAIR image.

MR physicists analyzed SPAIR sequences; their findings are summarized in Table S1 in the Supplementary Material. Among these sequences, SPAIR 4 consistently received the highest qualitative physicist grades (most preferred), whereas SPAIR 2 and 5 consistently received the lowest grades. Moderate-to-severe artifacts, including the herringbone (filtering), Gibbs ringing, zebra (3D phase aliasing), and partial volume artifacts, were consistently observed in SPAIR 2 and 5. One patient image acquired with SPAIR 4 had a bright blood vessels artifact. On average, SPAIR 4 and 2 had the highest percentage of slices that displayed observable amounts of anterior and posterolateral burnout, respectively. Conversely, SPAIR 5 had the lowest percentage of slices that displayed observable amounts of anterior and posterolateral burnout, but this is likely due to the increased number of slices in this sequence. Raw MR physicist feedback is provided in Appendix 2 in the Supplementary Material. The artifacts are illustrated in Figs. S3 and S4 in the Supplementary Material. Because of the consistently low-qualitative grade and persistence of severe artifacts among the patient images acquired with the sequences, SPAIR 2 and 5 were omitted from the primary analysis, which is subsequently described.

### 3.2 Quantitative Image Quality Analyses

#### 3.2.1 SNR and CNR measurements

The mean SNR of segmented fat was significantly higher in the nonsuppressed sequence compared with all of the SPAIR sequences. Thus each SPAIR sequence effectively suppressed the fat signal. Among the SPAIR sequences, SPAIR 1 had the largest mean fat SNR of 3.1, which was lower than the SNR of the target and OAR structures. The SNR for each structure was generally higher in the nonsuppressed sequence compared with the SPAIR sequences, as expected, although the SNR was significantly higher only for the parotid gland comparison.

The CNR between fat and the GTV were significantly higher in the SPAIR sequences compared with the nonsuppressed sequence. However, the CNR between fat and the pterygoid muscles was significantly higher in the nonsuppressed sequence compared with all of the SPAIR sequences. There were no significant differences in the CNR between fat and the parotid glands among all sequences.

Furthermore, the CNR between the muscle and the lymph nodes was significantly higher in the nonsuppressed sequence and the SPAIR 4 sequence compared with all other SPAIR sequences, and CNR between the muscle and parotid glands was significantly higher in the nonsuppressed sequence compared with all of the SPAIR sequences. There were no significant differences in the CNR between the muscle and the GTV among all sequences. Refer to Table S2 in the Supplementary Material for all SNR and CNR measurements.

#### 3.2.2 Conspicuity measurements

The conspicuity measurements for the structures in each sequence are depicted in Fig. S5 in the Supplementary Material. The median conspicuity was significantly lower in the nonsuppressed sequence compared with SPAIR 1 and 4 for the GTV and all of the SPAIR sequences for the lymph nodes and parotid glands. Conversely, the conspicuity of the pterygoid muscles was significantly higher in the nonsuppressed sequence compared with all of the SPAIR sequences. Among the SPAIR sequences, the conspicuity of SPAIR 3 was significantly lower compared with SPAIR 4 in all structures and SPAIR 1 in all structures except for the lymph nodes.

#### 3.2.3 Pairwise distance metrics

The medians of all pairwise DSC measurements between each segmentor for the structures in each sequence are depicted in Fig. S6 in the Supplementary Material. There was a large range of DSC values for the GTV in each sequence, and no significant differences were observed. The DSC of the lymph nodes was significantly higher in SPAIR 4 compared with the nonsuppressed image and other SPAIR images. In addition, the DSC in the parotid glands was significantly higher in all SPAIR sequences compared with the nonsuppressed sequence. Conversely, the DSC in the pterygoid muscles was significantly lower in all SPAIR sequences compared with the nonsuppressed sequence.

The medians of all pairwise HD measurements between each segmentor for the structures in each sequence are depicted in Fig. S7 in the Supplementary Material. These values followed the exact trend as the DSC measurements with regards to significant differences. Because smaller HD values represent more consistent segmentations, the trend between increased and decreased values was reversed relative to the DSC measurements (where larger DSC values represent more consistent segmentations).

### 3.3 Qualitative Image Quality Analyses

#### 3.3.1 Segmentor grading and comments

The segmentor image grades and the number of positive and negative comments for each sequence are illustrated in Figs. S8 and S9 in the Supplementary Material. SPAIR 1 and 3 received significantly higher median grades than the nonsuppressed sequence (SPAIR 4 versus Non-FS narrowly missed significance with p=0.08). SPAIR 1 received no negative comments for the GTV, and its segmentor comment metric for the GTV was significantly higher compared with the Non-FS sequence. Conversely, SPAIR 1 received no positive comments for the pterygoid muscles, and its segmentor comment metric for the pterygoid muscles was significantly lower compared with the Non-FS sequence. SPAIR 3 and 4 had net positive comments for all structures, and their segmentor comment metrics for the lymph nodes and parotid glands was significantly higher compared with the Non-FS sequence. However, because of the stringent scoring requirement described in Sec. 2.9, the rubric scores for this analysis were constant among sequence-structure pairs. Raw segmentor feedback is provided in Appendix 1 in the Supplementary Material.

### 3.4 Overall Sequence Scores

The results from the above analyses were analyzed according to the image analysis rubric. Specifically, they were formulated into metric scores for each structure-sequence pair and grouped into their respective categories (Tables S3–S6 in the Supplementary Material). The normalized category scores were then calculated and inputted into Table 2. The normalized category scores were summed and normalized to calculate the total scores for each sequence-structure pair. Among all of the sequences, the nonsuppressed sequence had the lowest total scores for the GTV, lymph nodes, and parotid glands but the highest score for the pterygoid muscles. SPAIR 1 and 4 had the highest total score for the GTV and parotid glands, and SPAIR 4 had the highest total score for the lymph nodes. When combining the total scores to calculate the combined total score, the nonsuppressed sequence scored the lowest, and SPAIR 4 scored the highest.

**Table 2:** Overall scores for each sequence from each analysis category and according to the image quality rubric. The normalized category scores for each structure from the SNR and CNR, conspicuity, pairwise distance, and segmentor categories were summed and normalized (4 = highest score) to calculate the total score and normalized total scores. The total score for each structure within a sequence was then summed across structures and renormalized to generate the combined total and combined normalized scores for each sequence.

Sequence	Non-FS				SPAIR 1				SPAIR 3				SPAIR 4			
Structure	GTV	LN	Par	Pty	GTV	LN	Par	Pty	GTV	LN	Par	Pty	GTV	LN	Par	Pty
SNR and CNR	1	2	4	2.5	3	2	2	2.5	3	2	2	2.5	3	4	2	2.5
Conspicuity	1.5	1	1	4	3.5	2.5	3.5	2.5	1.5	2.5	2	1	3.5	4	3.5	2.5
Pairwise distance	2.5	2	1	4	2.5	2	3	2	2.5	2	3	2	2.5	4	3	2
Segmentor	2.5	2.5	2.5	2.5	2.5	2.5	2.5	2.5	2.5	2.5	2.5	2.5	2.5	2.5	2.5	2.5
Total score	7.5	7.5	8.5	13	11.5	9	11	9.5	9.5	9	9.5	8	11.5	14.5	11	9.5
Normalized total score	1	1	1	4	3.5	2.5	3.5	2.5	2	2.5	2	1	3.5	4	3.5	2.5
Combined total score	36.5				41				36				46.5			
Combined normalized score	1				3				2				4			

Abbreviations: CNR, contrast-to-noise ratio; MR, magnetic resonance; Non-FS, non-suppressed; SNR, signal-to-noise ratio; and SPAIR, spectral attenuated inversion recovery.

## 4 Discussion

In this study, we developed several candidate SPAIR T2w sequences, reported here for the first time, so that fat-suppressed images could be incorporated into the treatment planning pipeline for patients with HNC treated on a Unity MR-Linac. We also developed a comprehensive image quality analysis platform to objectively score the sequences using a combination of quantitative and qualitative metrics. Using these metrics, the SPAIR sequence with the best combination of SNR, CNR, conspicuity, segmentation consistency, and qualitative segmentor assessment results for four structures—the GTV, metastatic lymph nodes, parotid glands, and pterygoid muscles—was identified. Both the optimized SPAIR sequence and the analysis platform can be used for clinical and research applications in radiation oncology.

After the five initial candidate SPAIR sequences were acquired in five patients, MR physicists performed an initial screening on the images to identify those with persistent image quality deficiencies. SPAIR 2 and 5 were consistently graded the lowest and produced numerous artifacts. Unlike the other SPAIR sequences, SPAIR 2 and 5 both used “through-plane” instead of “strong” free induction decay reduction. This parameter controls the crushing gradient that is responsible for attenuating residual magnetization during an echo train. Typically, this attenuation is achieved using in-plane gradients (such as in the “strong” option), but a research option for this parameter is “through-plane,” which uses the transverse gradient to attenuate the magnetization and reduces TE (also used in flow compensation). Insufficient magnetization attenuation can lead to stimulated echoes in an echo train and resultant “ringing-like” artifacts (separate from Gibbs), which appear as alternating lines of hyperintense and hypointense signals in an image. Examples of this artifact, which is also called a herringbone or filtering artifact, and other artifacts are shown in Fig. S6 in the Supplementary Material. Thus this free induction decay (FID) reduction parameter as an acceleration method is cautioned against for these types of sequences. No artifacts were identified in any of the other sequences (except for the “bright blood vessels” artifact in one of the SPAIR 4 patient images); this result was encouraging for the use of these SPAIR sequences in radiation oncology applications. In each SPAIR sequence, there was some degree of burnout in areas near tissue–air or tissue–bone interfaces away from the image isocenter, but this was expected due to $B_{0}$ inhomogeneity in these areas and did not impact segmentation. Because of these image quality deficiencies, SPAIR 2 and 5 were omitted from the subsequent primary analysis, which focused on the nonsuppressed sequence and SPAIR 1, 3, and 4.

The included metrics of the image analysis platform were carefully selected according to their applicability in HNC radiotherapy. For example, segmentation precision and qualitative comments were included in this analysis, although they may not be as necessary as other metrics for diagnostic imaging purposes. Metrics for patient motion were not analyzed in this study because of the use of immobilization devices in HNC radiotherapy. For the optimization of sequences for thoracic or abdominal imaging, these metrics would need to be considered in selecting the best sequence to use. The major benefit of this image analysis platform is that it can be easily customized through the addition, removal, or weighting of analysis metrics, depending on the application, and it can be generalized to any sequence (MRI or another modality) for any number of structures. For example, if users desired to give the results of the segmentor assessment twice as much weight as the other analysis categories, they could simply double the normalized category score of this analysis category when calculating the total scores and updated total scores. Alternatively, if they wanted the outcomes of the GTV and lymph node target structure analyses to be given twice as much weight as the outcomes of the parotid gland and pterygoid muscle analyses segmentations, they could simply double the scores of the former when calculating the combined total score. It is up to the individual user or institution to decide on how these weights should be implemented. A similar multicategory grading system to the one in this study was described by Dimaridis et al.,^52^ in which several studies in the field of optoacoustic imaging were reviewed and graded based on author-defined criteria of the study quality. The criteria had multiple subgroups and were summed and normalized in a similar manner as this study. The authors stated that this scoring system was developed to be “applicable to works with a broad range of purposes, as well as to emphasize the importance of evaluation with human subjects.” However, the criteria were limited to qualitative assessments due to the nature of the analysis. Miéville et al.^53^ used multiple objective criteria with a subjective grading from two radiologists to test the effects of an image denoising reconstruction algorithm, though they were not combined in one overall score.

From the comprehensive analysis, the nonsuppressed sequence had lower scores than each SPAIR sequence for the GTV, lymph nodes, and parotid gland structures. Interestingly, the opposite was true for the pterygoid muscle structures, for which the nonsuppressed sequence had scores superior to those for each of the SPAIR sequences. This result was likely due to the fact that muscle appears hypointense in T2w sequences, so its contrast was further reduced after the signal from fat was suppressed. Among the SPAIR sequences, SPAIR 1 and 4 scored higher or equivalent to SPAIR 3 in every category for all structures. For the GTV, parotid glands, and pterygoid muscles, SPAIR 1 and 4 performed very similarly for the various metrics. This was not too surprising considering that the sequence parameters were very similar between the two sequences except in regard to the refocusing angle (SPAIR 1, 40 deg; SPAIR 4, 55 deg), ${\mathrm{TE}}_{\mathrm{equiv}}$ (SPAIR 1, 93 ms; SPAIR 4, 107 ms), and (SPAIR 1, 1600 ms; SPAIR 4, 1400 ms). These sequences also had larger oversampling factors and smaller water-fat shifts than the remaining SPAIR sequences. It should be noted that SPAIR 4 was consistently the best performing sequence for the lymph node structures, which could perhaps be explained by slight differences in T2 weighting. Furthermore, the acquisition time for SPAIR 4 was nearly 1 min less than that for SPAIR 1. Users may want to test both sequences to determine which fits their personal preferences, but we officially recommend that SPAIR 4 be used for HNC radiotherapy treatment planning. Because extended FOV scans can be helpful for identifying lower neck lymph nodes in the head and neck region, we attempted to create an extended FOV sequence (SPAIR 5) with an equivalent acquisition time as the other SPAIR iterations. However, the acceleration techniques required for this acceleration resulted in severe imaging artifacts. Thus we recommend extending the FOV of SPAIR 4, rather than using SPAIR 5, if lower neck nodes need to be imaged. Adding the extra 50 slices to SPAIR 4 to match the FOV of SPAIR 5 would result in an increased acquisition time of 2:30.

Additional criteria, other than the absence of severe artifacts, should be met before MR images are used for radiation oncology applications. High geometric accuracy is arguably the most important criterion to ensure accurate target coverage during treatment delivery. System-based geometric distortion on the Unity MR-Linac has been extensively investigated and is reported to be ∼1 to 2 mm for a 350-mm diameter spherical volume, which still results in treatment plan accuracy within recommended tolerances in phantoms and patients.^19^^,^^54^^,^^55^ We observed similar results in the nonsuppressed and SPAIR 1 and 4 sequences using an Elekta geometric distortion phantom (data provided in Table S7 and Fig. S10 in the Supplementary Material). Furthermore, there were no significant differences in the distortion measurements between the nonsuppressed sequence (currently used clinically) and either SPAIR sequence. Geometric distortion also manifests from susceptibility artifacts, which can occur in the head and neck region due to dental implants, bone, and air that cause local changes in the magnetic field. Imaging at a low magnetic field and using spin echo rather than gradient echo sequences are the most effective methods for mitigating this artifact. Because all of the sequences in this study are fast spin echo and acquired at the same magnetic field, they all possess similar levels of susceptibility artifacts. Additionally, high-resolution images are needed for the accurate delineation of structures. Each of the SPAIR sequences had an isotropic reconstructed voxel size of 1 mm or less (except for SPAIR 5, which had a larger through-plane field of view and resultant voxel size). Finally, the target structures and OARs need to be visible in the images for accurate and consistent segmentation. The SNR, CNR, conspicuity, pairwise distance, and segmentor analyses in our study all demonstrated that representative structures were more detectible with the SPAIR sequences than with the nonsuppressed T2w sequence.

Several studies have evaluated one or more fat suppression methods for head and neck diagnostic imaging.^56^^–^^59^ A few studies further compared various fat-suppressed techniques for the head and neck region. Gaddikeri et al.^33^ compared the Dixon and STIR techniques in T2w images as well as the Dixon and SPIR techniques in postcontrast T1w images. The Dixon method outperformed both the STIR and the SPIR techniques in terms of signal intensity and reader-graded image quality. In a series of publications, Ma et al.^34^^,^^60^ demonstrated that, in the head and neck region, a triple-echo Dixon technique suppressed the fat signal to a higher degree and more uniformly than did the CHESS and alternative Dixon techniques. These results were corroborated by Wendl et al.^61^ Kawai et al.^62^ compared coronal STIR and axial SPIR techniques for the detection of metastatic lymph nodes in HNC. The authors stated that both techniques performed comparably, although the STIR sequence was shorter and had fewer susceptibility artifacts than did the SPIR sequence.

Dixon-based sequences are strong candidates for fat suppression. However, there are currently no Dixon sequences available to clinical users of the Unity MR-Linac, and only 2D, T2w, Dixon sequences are available to select research users. 2D sequences are generally unusable for treatment planning purposes for two reasons: the lack of precision when delineating structures and the logistical inability to import 2D images into the treatment planning system. Because we wanted to develop and optimize a sequence that could be broadly disseminated to other Unity users without the need for a research patch, we opted not to pursue the development of a 3D, T2w, Dixon sequence. Thus for the time being, SPAIR is the optimal clinically available fat suppression technique for HNC treatment planning on the Unity MR-Linac. If 3D Dixon sequences are broadly enabled on the Unity, then we plan to compare an optimized 3D Dixon sequence with the optimized SPAIR sequence presented in this paper. It should be noted that, in a study by Huijgen et al.^63^ that compared Dixon and SPAIR sequences for musculoskeletal tumor imaging, there were minimal differences among the image quality metrics for the sequences, except in terms of fat suppression homogeneity, which was superior in the Dixon sequences. In addition, the Dixon sequences performed noticeably better in areas with large $B_{0}$ inhomogeneities, which suggests that future investigations of Dixon sequences in HNC treatment planning are warranted. Finally, once compressed sensing is clinically available for the Unity MR-Linac, the iterative reconstruction of undersampled data could accelerate sequences by up to 40%, and it would be worthwhile to use the framework outlined in this paper to reevaluate accelerated SPAIR sequences.

Compared with diagnostic systems, the imaging quality of the MR-Linac is slightly compromised to allow for the delivery of megavoltage beams. For example, the gradient coils are split at isocenter to allow for the entry of a beam up to 22 cm wide, but this makes it susceptible to eddy currents and gradient nonlinearities. Eddy currents can lead to unwanted time-varying gradients as well as $B_{0}$ inhomogeneities and were shown to be the main source of distortion around isocenter rather than gradient nonlinearities.^64^^,^^65^ Although these effects are more pronounced in sequences with fast-switching gradients, such as EPI and diffusion scans, turbo spin echo sequences are still susceptible to shading artifacts. And although eddy currents can affect alternative fat suppression techniques such as CHESS and SPIR sequences, the adiabatic inversion pulse used in SPAIR sequences is highly insensitive to local $B_{1}$ inhomogeneities and frequency shifts induced by eddy currents. This is also beneficial to mitigating any $B_{1}$ inhomogeneities produced by the radiolucent quadrature body coil used to transmit RF power (the anterior and posterior array surface coils are receive-only and do not contribute to $B_{1}$ inhomogeneities). This quadrature body coil was designed to maintain reproducibility and minimize dosimetry modulation of treatment plans.^66^^,^^67^ However, image quality with this coil is impacted because it cannot be positioned as close to the anatomy as four-channel flex coils commonly used in head and neck MR simulations, nor can it produce the same amount of SNR as 64-channel head and neck volume coils that are often used in diagnostic settings. Furthermore, the maximum gradient strength and slew rate of the MR-Linac gradient coils is lower than what is seen in typical diagnostic and simulation MR scanners, resulting in slower gradient encoding.^65^ Although this does have a diminishing effect on SNR, T2-weighted sequences are not otherwise significantly impacted because they do not utilize short TE values. Finally, even though the gantry hardware can rotate around the MR-Linac bore and operate during image acquisition, it was demonstrated that it minimally impacts image quality.^19^^,^^65^

A limitation of this study was that only a few patients were included in the analysis, although for initial sequence development and optimization studies, this is not too unusual because the time required to manually segment multiple structures per image for multiple images per patient limits the number of patients that can realistically be included in one study. This limitation was mediated by our inclusion of multiple independent segmentors. Further multiinstitutional validation of these sequences within the MR-Linac Consortium would add power to these results. Furthermore, investigating the usefulness of these sequences for autosegmentation purposes would greatly increase the number of available segmentations. A perceived limitation to this study could be that the scores on the rubric were not substantially different between the SPAIR sequences. This is due to the requirement that sequences with higher rubric scores must have significantly greater (p<0.05) metric values than all sequences with lower rubric scores. In the statistical analysis, the p-values were corrected for multiple comparisons, which further results in more stringent criteria. A final limitation to the study was that the front-end treatment planning effects (segmentations), but not the downstream dosimetric implications, were investigated. But because there was no ground-truth segmentation for the investigated structures, only relative differences in downstream doses from differing segmentations would have been inferred. A study designed to investigate the clinical implications of using or excluding an optimized fat-suppressed sequence during the treatment planning process would be of great interest.

## 5 Conclusions

In this study, we optimized a 3D, T2w SPAIR sequence for HNC treatment planning on the Unity 1.5 T MR-Linac. This sequence and the other candidate sequences are available for download for use on other Unity devices. We also developed a robust image quality analysis platform that can be customized and generalized to any type of image optimization. This is the first study to report data on fat suppression methods in the head and neck using the Unity MR-Linac and to provide a working sequence for fat suppression. Furthermore, this is the first study to report a SPAIR T2w image quality assessment for primary and metastatic structures in HNC. We believe that HNC treatment planning and subsequent treatment outcomes can be improved through the use of this sequence.

## Data Availability

Research data for this study are available in FigShare (https://doi.org/10.6084/m9.figshare.20140184).
