# Supplementary material for: Development and implementation of optimized endogenous contrast sequences for delineation in adaptive radiotherapy on a 1.5T MR-linear-accelerator: a prospective R-IDEAL stage 0-2a quantitative/qualitative evaluation of in vivo site-specific quality-assurance using a 3D T2 fat-suppressed platform for head and neck cancer
Source: J Med Imaging (Bellingham). 2023 Nov 6;10(6):065501. doi: 10.1117/1.JMI.10.6.065501 (PMC10627232; doi:10.1117/1.JMI.10.6.065501)
Supplement: Supplementary file 1 [file JMI_010_065501_SD001.pdf]

Supplementary Materials

Supplementary Tables

Pulse sequence	Qualitative score	Slices with anterior burnout	Slices with posterolateral burnout	Observed artifacts
**SPAIR 1**	4<br>(3.00 – 4.00)	27.5%<br>(25.0% – 45.0%)	17.5%<br>(15.0% – 25.0%)	None
**SPAIR 2**	2<br>(1.00 – 2.00)	28.8%<br>(18.8% – 38.8%)	25.0%<br>(22.5% – 30.0%)	Herringbone<br>Gibbs ringing<br>Zebra artifact
**SPAIR 3**	3.25<br>(3.00 – 4.25)	27.5%<br>(21.3% – 37.5%)	20.0%<br>(17.5% – 32.5%)	None
**SPAIR 4**	5<br>(4.75 – 5.00)	32.5%<br>(20.6% – 35.0%)	17.5%<br>(17.5% – 25.0%)	Bright blood vessels
**SPAIR 5**	1.25<br>(1.00 – 2.00)	24.0%<br>(15.0% – 34.0%)	17.0%<br>(12.0% – 22.0%)	Herringbone<br>Gibbs ringing<br>Partial volume

Table S1: MR physicists' assessments of the non-suppressed and spectral presaturation with inversion recovery (SPAIR) sequences. A higher grade corresponds to a more preferred sequence. Numerical values are expressed as medians and interquartile ranges. Artifacts mentioned by either physicist on any patient image is listed.

Pulse sequence	Fat SNR	Structure	Structure SNR	Structure-fat CNR	Structure-muscle CNR
**Non-FS**	**20.6 ± 3.9***	GTV	16.1 ± 4.9	4.5 ± 4.0	7.7 ± 4.4
		Lymph nodes	20.3 ± 5.8	3.2 ± 2.9	**11.6 ± 5.3***
		Parotid glands	**15.9 ± 2.8***	4.7 ± 2.1	**7.6 ± 1.7***
		Pterygoid muscles	8.5 ± 1.6	**12.1 ± 2.2***	
**SPAIR 1**	3.1 ± 1.1	GTV	16.5 ± 6.2	**13.4 ± 5.4***	7.7 ± 1.7
		Lymph nodes	12.5 ± 4.5	9.2 ± 4.2	5.9 ± 2.5
		Parotid glands	10.7 ± 4.6	7.5 ± 3.8	1.8 ± 1.1
		Pterygoid muscles	9.0 ± 4.4	5.9 ± 3.7	
**SPAIR 3**	2.4 ± 0.8	GTV	13.7 ± 1.6	**11.3 ± 1.0***	7.3 ±1.9
		Lymph nodes	10.1 ± 4.0	7.8 ± 4.4	6.2 ± 2.7
		Parotid glands	7.8 ± 1.6	5.5 ± 1.0	1.5 ± 0.8
		Pterygoid muscles	6.5 ± 2.2	4.1 ± 1.7	
**SPAIR 4**	2.8 ± 0.8	GTV	15.2 ± 4.0	**12.5 ± 3.5***	8.4 ± 2.5
		Lymph nodes	16.2 ± 7.0	13.3 ± 6.7	**10.3 ± 3.8***
		Parotid glands	9.4 ± 3.0	6.6 ± 2.6	2.5 ± 1.4
		Pterygoid muscles	7.0 ± 2.2	4.3 ± 1.9	

Table S2: Signal-to-noise ratio (SNR) and contrast-to-noise ratio (CNR) measurements of the gross primary tumor volume (GTV), lymph nodes, parotid glands, and pterygoid muscles in the non-suppressed (Non-FS) and spectral presaturation with inversion recovery (SPAIR) sequences. For each sequence, SNR of fat was calculated to quantify the degree of fat suppression. The SNR was also calculated for each structure within the sequences, as was the CNR relative to the fat and muscle signals. Values are presented as means ± standard deviations. Values for a specific sequence-structure pair that are denoted with an asterisk are significantly greater ($P<.05$) than all values for the structure in the column that are not denoted with an asterisk.

Sequence	Non-FS				SPAIR 1				SPAIR 3				SPAIR 4			
**Structure**	GTV	LN	Par	Pty	GTV	LN	Par	Pty	GTV	LN	Par	Pty	GTV	LN	Par	Pty
**Fat SNR**	1				3				3				3			
**Structure SNR**	2.5	2.5	4	2.5	2.5	2.5	2	2.5	2.5	2.5	2	2.5	2.5	2.5	2	2.5
**CNR-fat**	1	2.5	2.5	4	3	2.5	2.5	2	3	2.5	2.5	2	3	2.5	2.5	2
**CNR-mus**	2.5	3.5	4	2.5	2.5	1.5	2	2.5	2.5	1.5	2	2.5	2.5	3.5	2	2.5
**Total score**	7	9.5	11.5	10	11	9.5	9.5	10	11	9.5	9.5	10	11	11.5	9.5	10
**Normalized score**	1	2	4	2.5	3	2	2	2.5	3	2	2	2.5	3	4	2	2.5

Table S3: Signal-to-noise ratio (SNR) and contrast-to-noise ratio (CNR) metric scores.

Abbreviations: GTV, gross primary tumor volume; LN, lymph nodes; mus, muscle; Non-FS, non-suppressed; Par, parotid glands; Pty, pterygoid muscles; SPAIR, spectral presaturation with inversion recovery.

Sequence	Non-FS				SPAIR 1				SPAIR 3				SPAIR 4			
**Structure**	GTV	LN	Par	Pty	GTV	LN	Par	Pty	GTV	LN	Par	Pty	GTV	LN	Par	Pty
**Conspicuity**	1.5	1	1	4	3.5	2.5	3.5	2.5	1.5	2.5	2	1	3.5	4	3.5	2.5
**Total score**	1.5	1	1	4	3.5	2.5	3.5	2.5	1.5	2.5	2	1	3.5	4	3.5	2.5
**Normalized score**	1.5	1	1	4	3.5	2.5	3.5	2.5	1.5	2.5	2	1	3.5	4	3.5	2.5

Table S4: Conspicuity metric scores.

Abbreviations: GTV, gross primary tumor volume; LN, lymph nodes; Non-FS, Non-suppressed; Par, parotid glands; Pty, pterygoid muscles; SPAIR, spectral presaturation with inversion recovery.

Sequence	Non-FS				SPAIR 1				SPAIR 3				SPAIR 4			
**Structure**	GTV	LN	Par	Pty	GTV	LN	Par	Pty	GTV	LN	Par	Pty	GTV	LN	Par	Pty
**DSC**	2.5	2	1	4	2.5	2	3	2	2.5	2	3	2	2.5	4	3	2
**HD**	2.5	2	2	4	2.5	2	3	2	2.5	2	3	2	2.5	4	3	2
**Total score**	5	4	3	8	5	4	6	4	5	4	6	4	5	8	6	4
**Normalized score**	2.5	2	1	4	2.5	2	3	2	2.5	2	3	2	2.5	4	3	2

Table S5: Pairwise distance metric scores.

Abbreviations: DSC, Dice similarity coefficient; GTV, gross primary tumor volume; HD, Hausdorff distance; LN, lymph nodes; Non-FS, non-suppressed; Par, parotid glands; Pty, pterygoid muscles; SPAIR, spectral presaturation with inversion recovery.

Sequence	Non-FS				SPAIR 1				SPAIR 3				SPAIR 4			
**Structure**	GTV	LN	Par	Pty	GTV	LN	Par	Pty	GTV	LN	Par	Pty	GTV	LN	Par	Pty
**Segmentor score**	2.5				2.5				2.5				2.5			
**Segmentor comment**	2.5	2.5	2.5	2.5	2.5	2.5	2.5	2.5	2.5	2.5	2.5	2.5	2.5	2.5	2.5	2.5
**Total score**	5	5	5	5	5	5	5	5	5	5	5	5	5	5	5	5
**Normalized score**	2.5	2.5	2.5	2.5	2.5	2.5	2.5	2.5	2.5	2.5	2.5	2.5	2.5	2.5	2.5	2.5

Table S6: Segmentor grading and comments metric scores.

Abbreviations: GTV, gross primary tumor volume; LN, lymph nodes; Non-FS, non-suppressed Par, parotid glands; Pty, pterygoid muscles; SPAIR, spectral presaturation with inversion recovery.

Sequence	Non-FS	SPAIR 1	SPAIR 4
**ML distortion (mm)**	1.03 ± 0.58	1.60 ± 0.56	1.65 ± 0.60
**AP distortion (mm)**	0.90 ± 0.43	0.72 ± 0.31	0.70 ± 0.30
**SI distortion (mm)**	1.11 ± 0.40	1.01 ± 0.37	0.97 ± 0.37

Table S7: Geometric distortion measurements along each anatomic plane measured at 300 mm diameter spherical volume (DSV). Measurements were made using the Elekta geometric distortion phantom provided with the Unity magnetic resonance linear accelerator. These values represent the 98th percentile values ± the standard deviations and are less than 2 mm, which demonstrates that there was no significant geometric distortion among the spectral presaturation with inversion recovery (SPAIR) sequences. When comparing the distributions using a 1-way analysis of variance test, there were no statistically significant differences between the SPAIR sequences or between either the SPAIR 1 or SPAIR 4 sequence and the non-suppressed, T2-weighted sequence that is conventionally used in the clinic. Refer to Figure S5 for an illustration of the distortion distributions along each anatomic plane.

Abbreviations: AP, anterior/posterior; ML, medial/lateral; Non-FS, non-suppressed; SI, superior/inferior.

Supplementary Figures

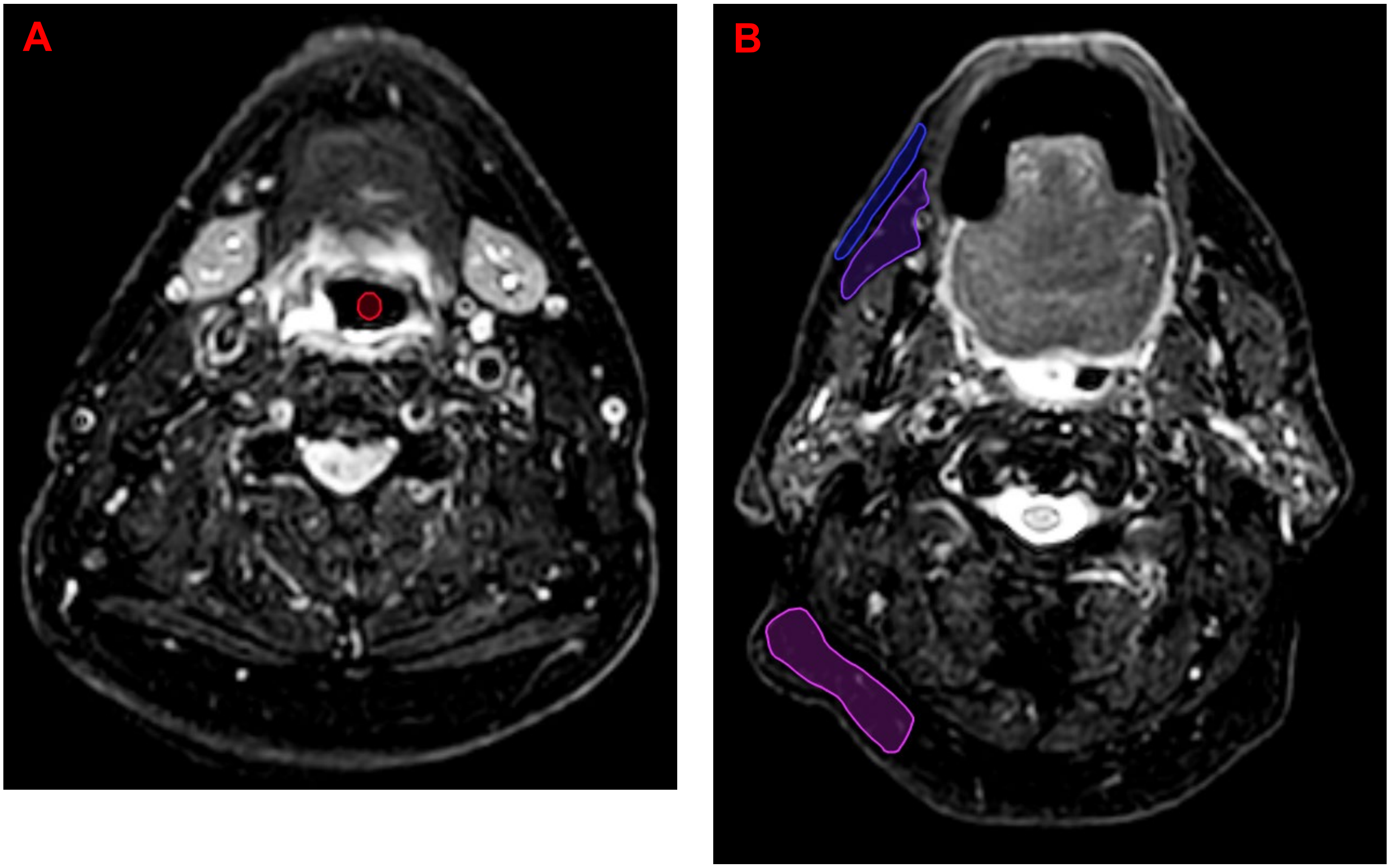

Figure S1: Representative noise (**A**) and fat (**B**) segmentations that are used in contrast-to-noise (CNR) measurements. The noise segmentation (red circle) is placed in the air-filled trachea and extends for 10 image slices. The fat segmentations are drawn in 3 separate areas of neck and cheek fat on one image slice. Care was taken to consistently draw these segmentations on the same slice(s) for each sequence.

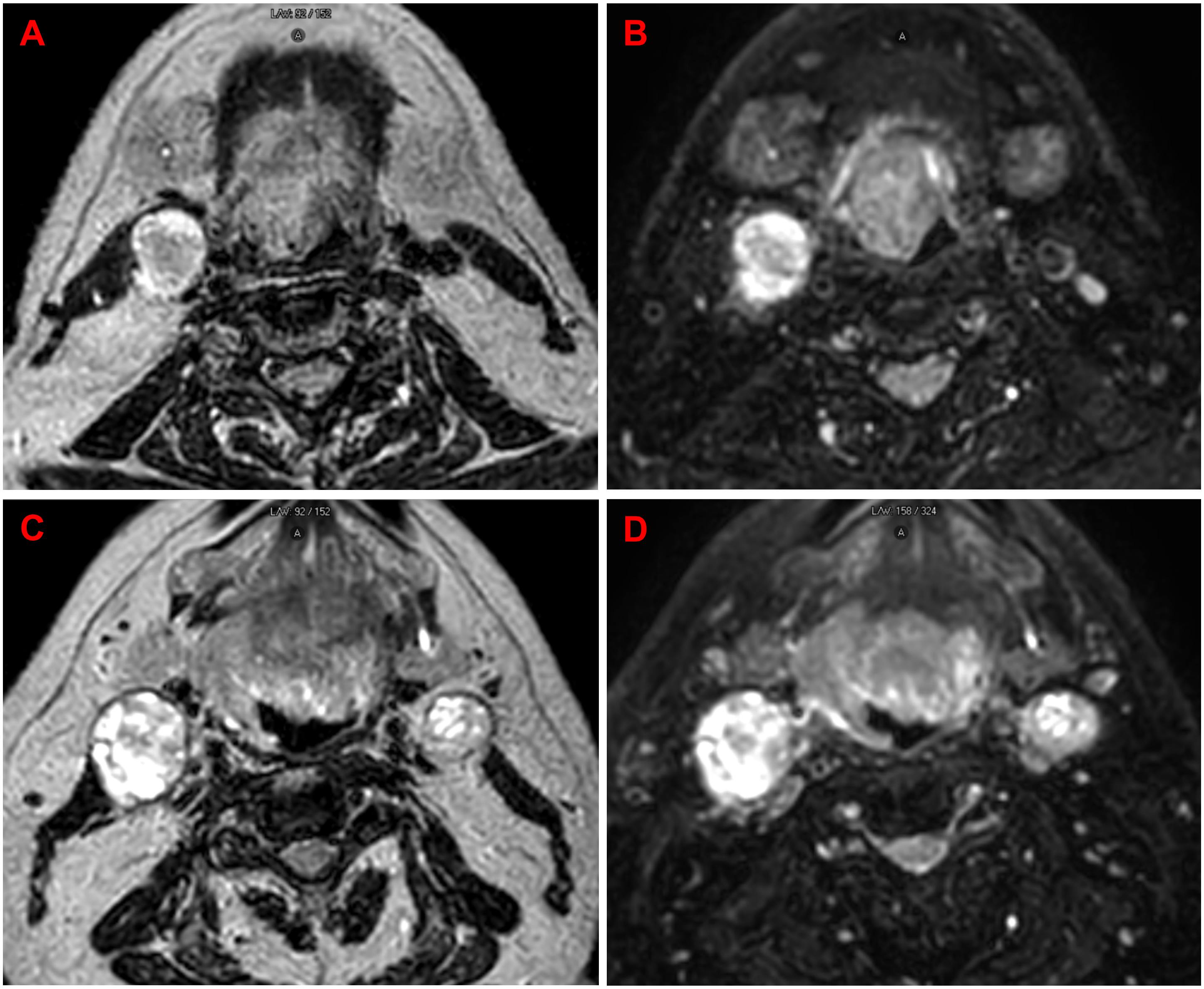

Figure S2: Representative (**A** and **C**) non-suppressed, T2-weighted and (**B** and **D**) spectral attenuated inversion recovery (SPAIR) T2-weighted image slices without visible segmentations in a patient with head and neck cancer. These images are acquired using the SPAIR 4 sequence. Refer to Fig. 3 for segmentations annotating the gross primary tumor volume and metastatic lymph nodes.

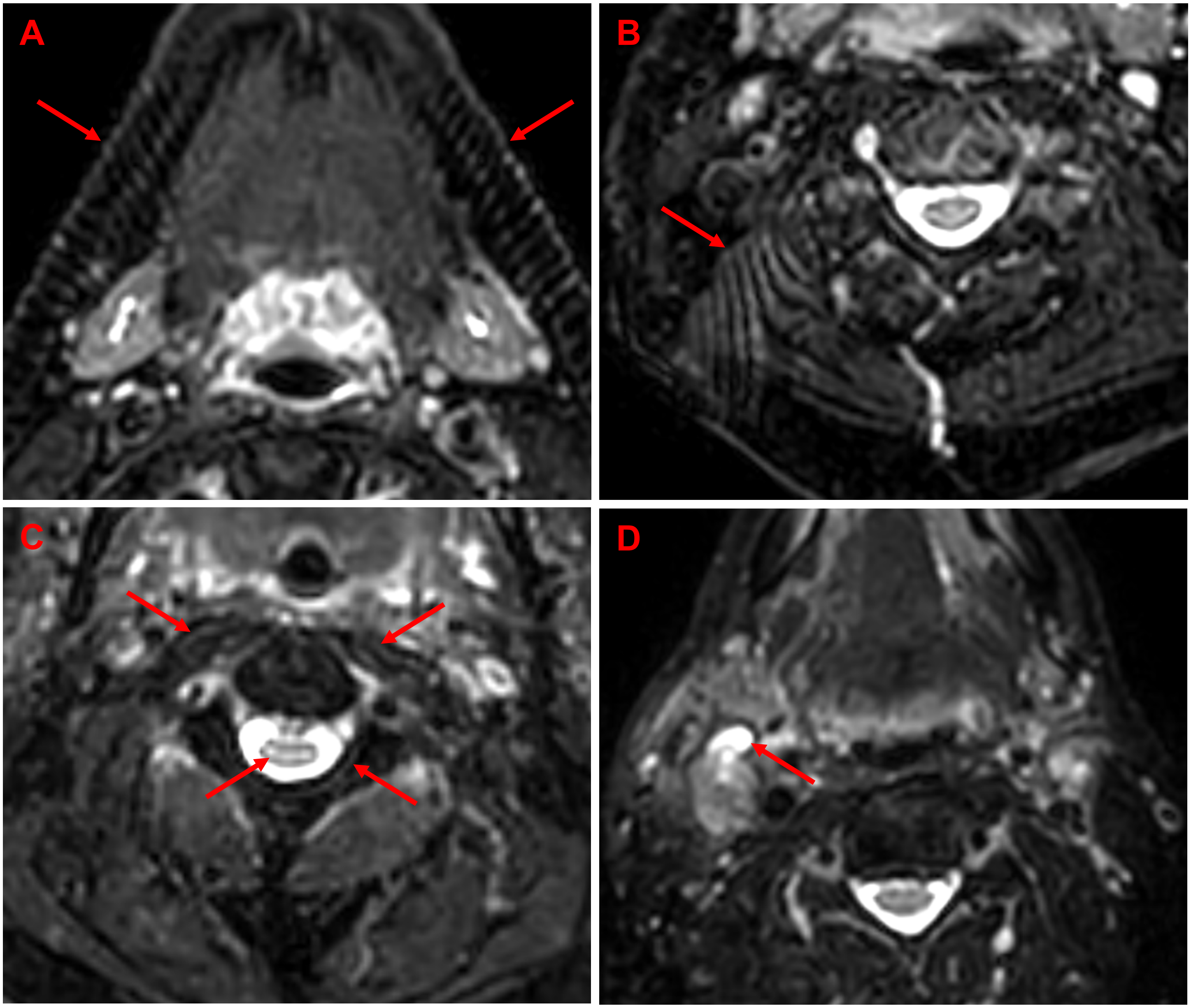

Figure S3: Representative examples of the artifacts that were observed in the spectral attenuated inversion recovery (SPAIR) images. The red arrows indicate the artifacts. (**A**) The herringbone (filtering) artifact in the jaw. (**B**) the zebra (3-dimensional phase aliasing) artifact in the posterior area of the head. (**C**) The Gibbs ringing artifact around the spinal cord and in other areas with abrupt intensity changes. (**D**) A bright blood vessel artifact, which is not present on the contralateral side. This was the only patient in which this artifact was observed.

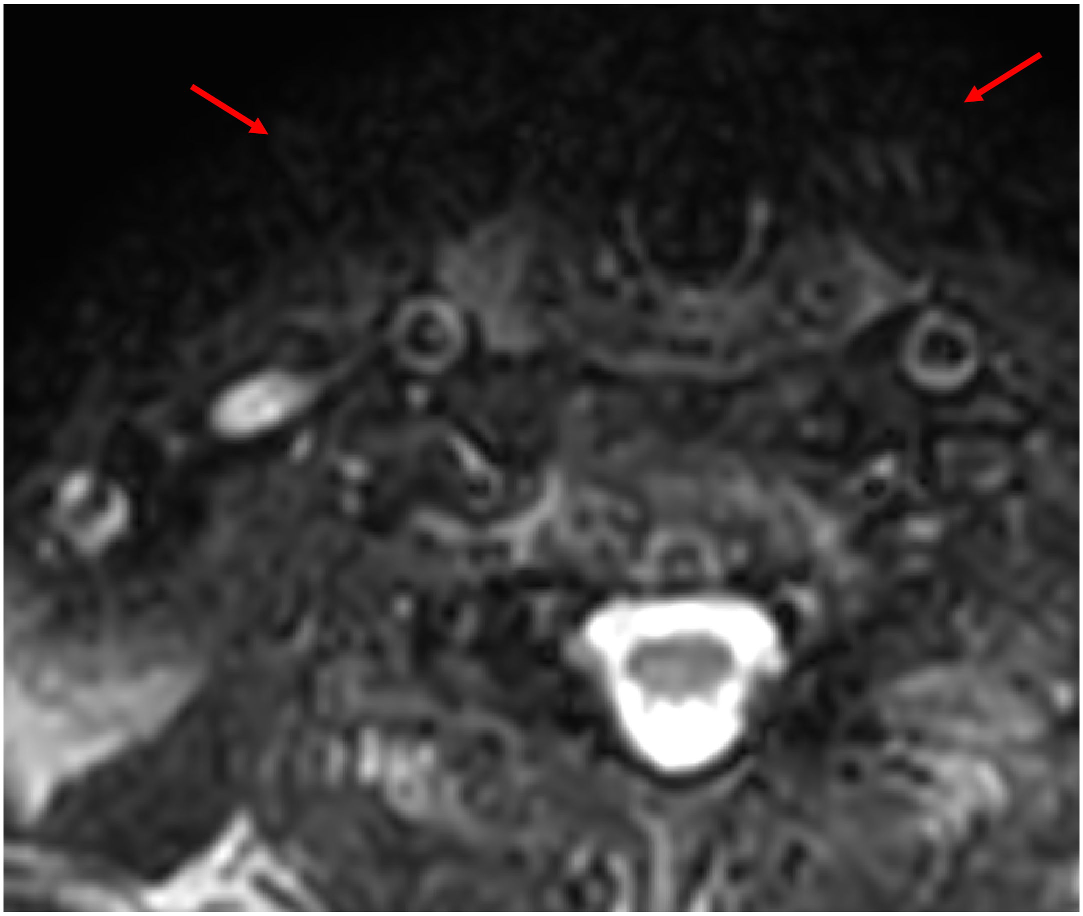

Figure S4: An example of burnout (denoted by red arrows), which was observed in some capacity in each of the spectral attenuated inversion recovery (SPAIR) sequences. The homogeneous hypointensity is a result of improper fat suppression in areas of B_0 inhomogeneity. It is typically seen in the anterior and posterolateral areas of the image, which are far from image isocenter of and around tissue-air or tissue-bone interfaces.

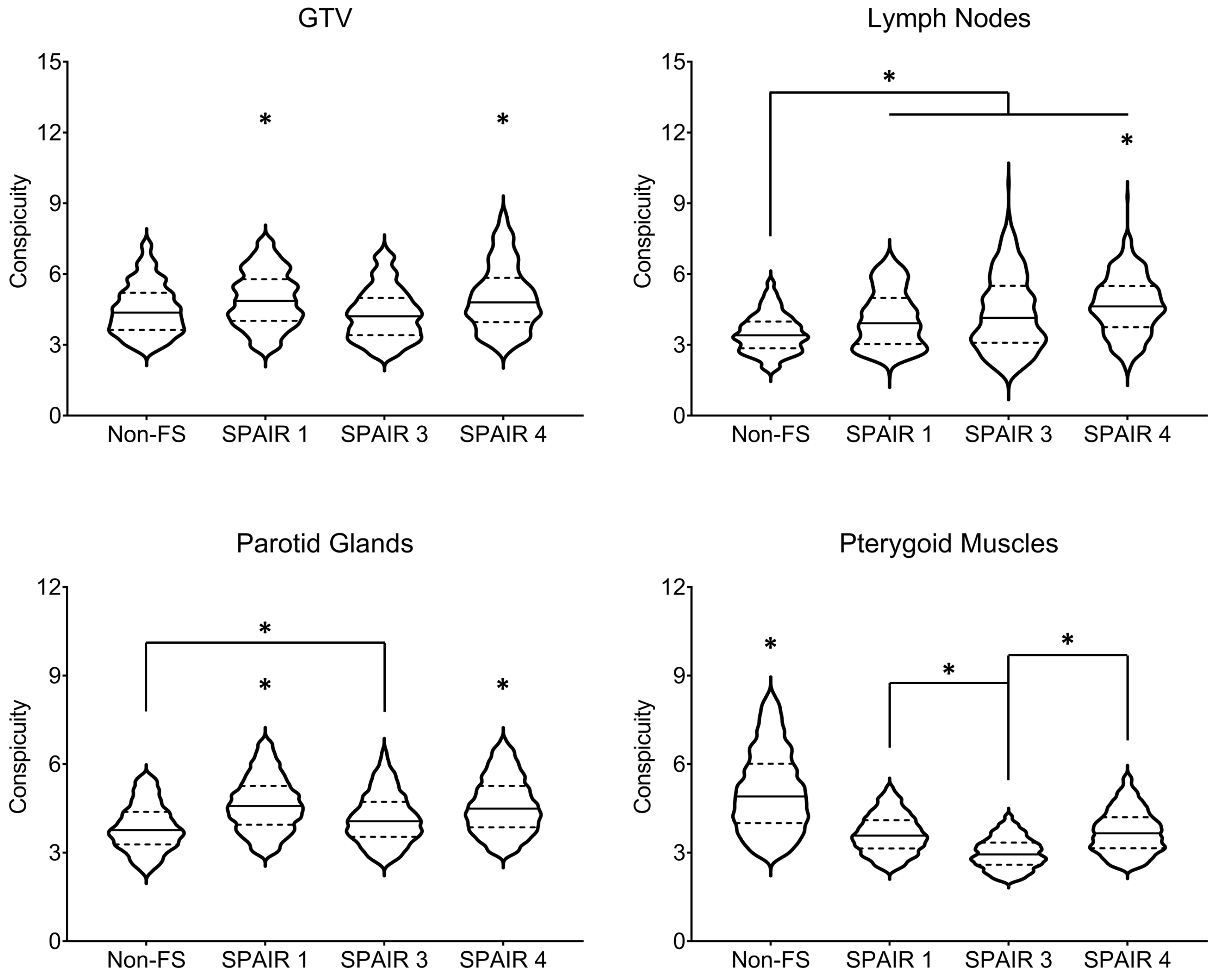

Figure S5: Conspicuity measurements of the gross primary tumor volume (GTV), lymph nodes, parotid glands, and pterygoid muscles in the non-suppressed and spectral attenuated inversion recovery (SPAIR) sequences. Solid lines represent the median values of the distributions, and dashed lines represent the limits of the interquartile range. Unless specifically annotated, all entries denoted with an asterisk* are significantly greater (*P*<.05) than all entries that are not denoted with an asterisk.

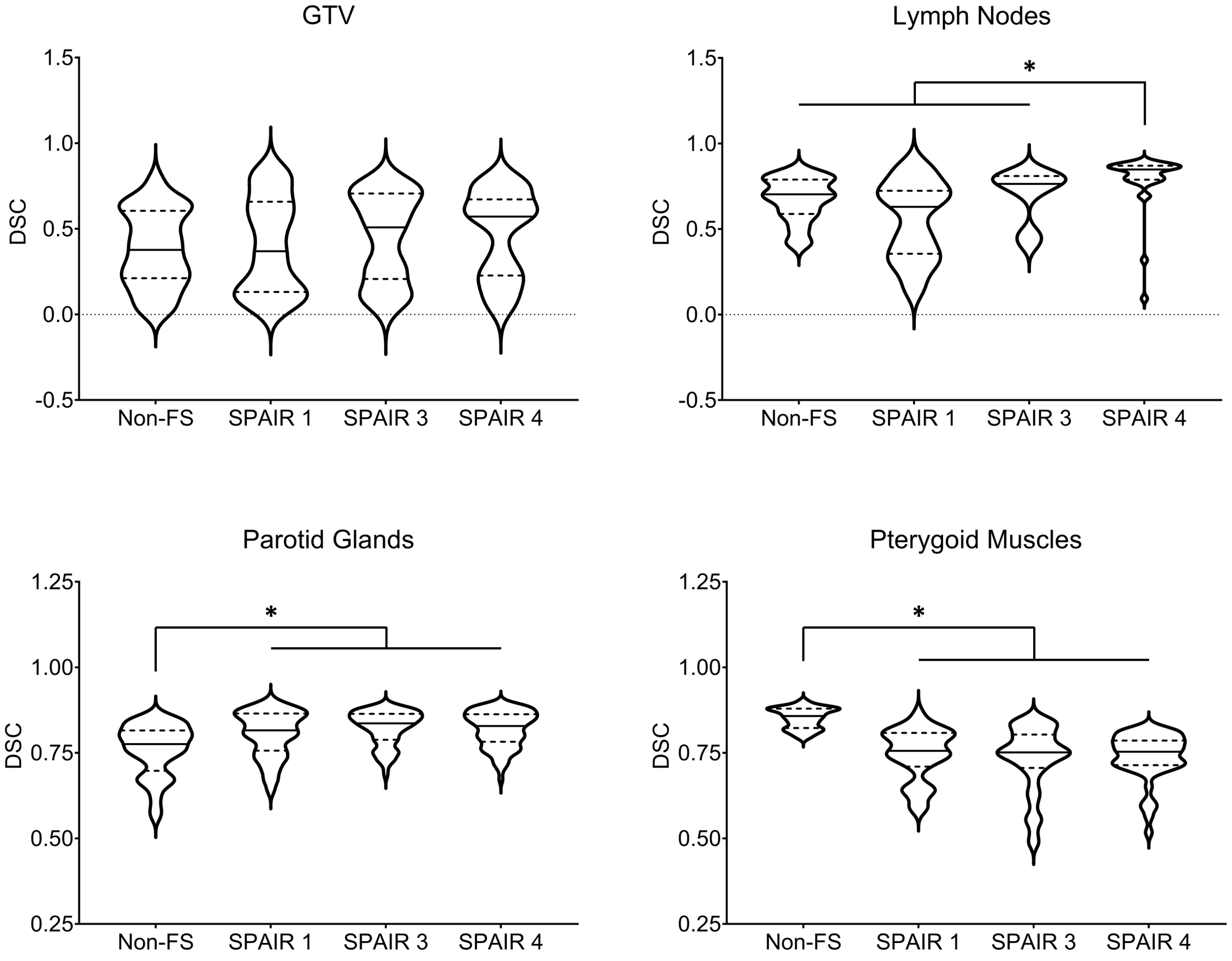

Figure S6: Dice similarity coefficient (DSC) measurements of the gross primary tumor volume (GTV), lymph nodes, parotid glands, and pterygoid muscles in the non-suppressed and spectral attenuated inversion recovery (SPAIR) sequences. Solid lines represent the median values of the distributions, and dashed lines represent the limits of the interquartile range. An asterisk* indicates a significant difference ($P<.05$). Although some of the violin plot distributions extend below 0 and above 1, all DSC values were positive and between 0 and 1.

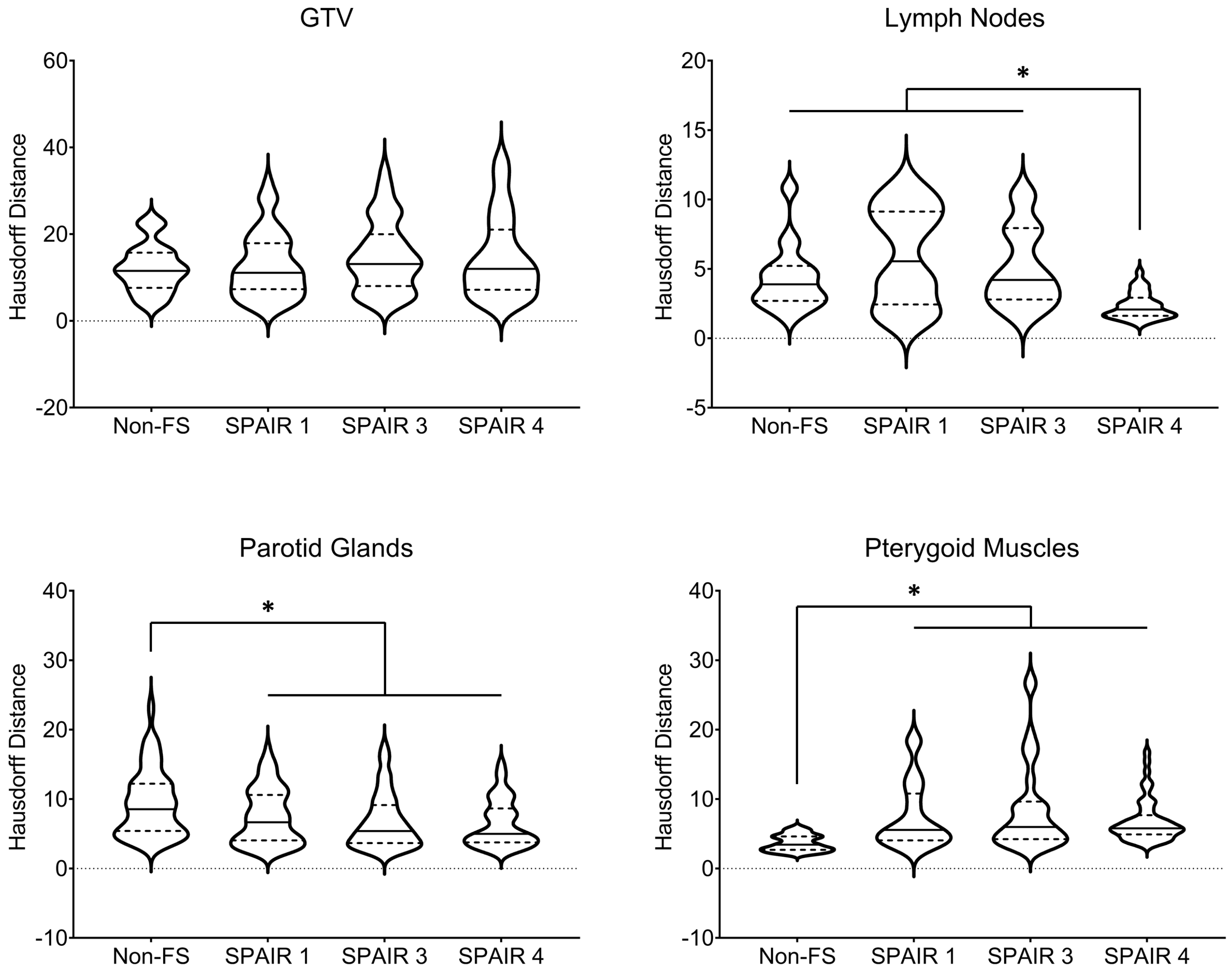

Figure S7: Hausdorff distance measurements of the gross primary tumor volume (GTV), lymph nodes, parotid glands, and pterygoid muscles in the non-suppressed and spectral attenuated inversion recovery (SPAIR) sequences. Solid lines represent the median values of the distributions, and dashed lines represent the limits of the interquartile range. An asterisk* indicates a significant difference (*P*<.05). Although some of the violin plots extend below 0, all Hausdorff distance values were positive.

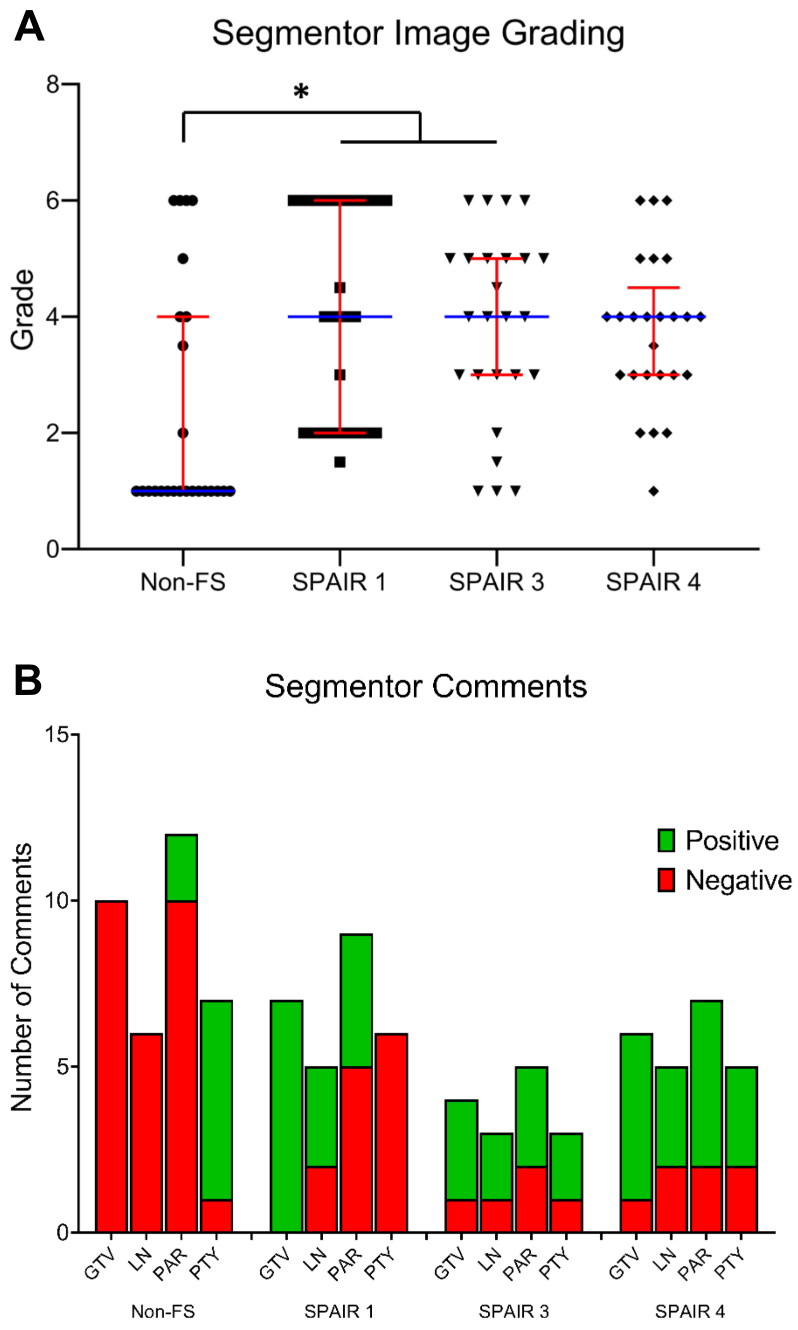

Figure S8: Segmentor image grading and comments on the gross primary tumor volume (GTV), lymph nodes (LN), parotid glands (PAR), and pterygoid muscles (PTY) in the non-suppressed and spectral attenuated inversion recovery (SPAIR) sequences. In the plot of segmentor grades (**A**), median grades are depicted by horizontal blue lines, and interquartile ranges are depicted by vertical red lines. Each point represents a segmentor grade for a patient image. A higher grade corresponds to a more preferred sequence. The number of segmentor comments classified as positive and negative are illustrated in stacked bar plots (**B**). An asterisk* indicates a significant difference (P<.05).

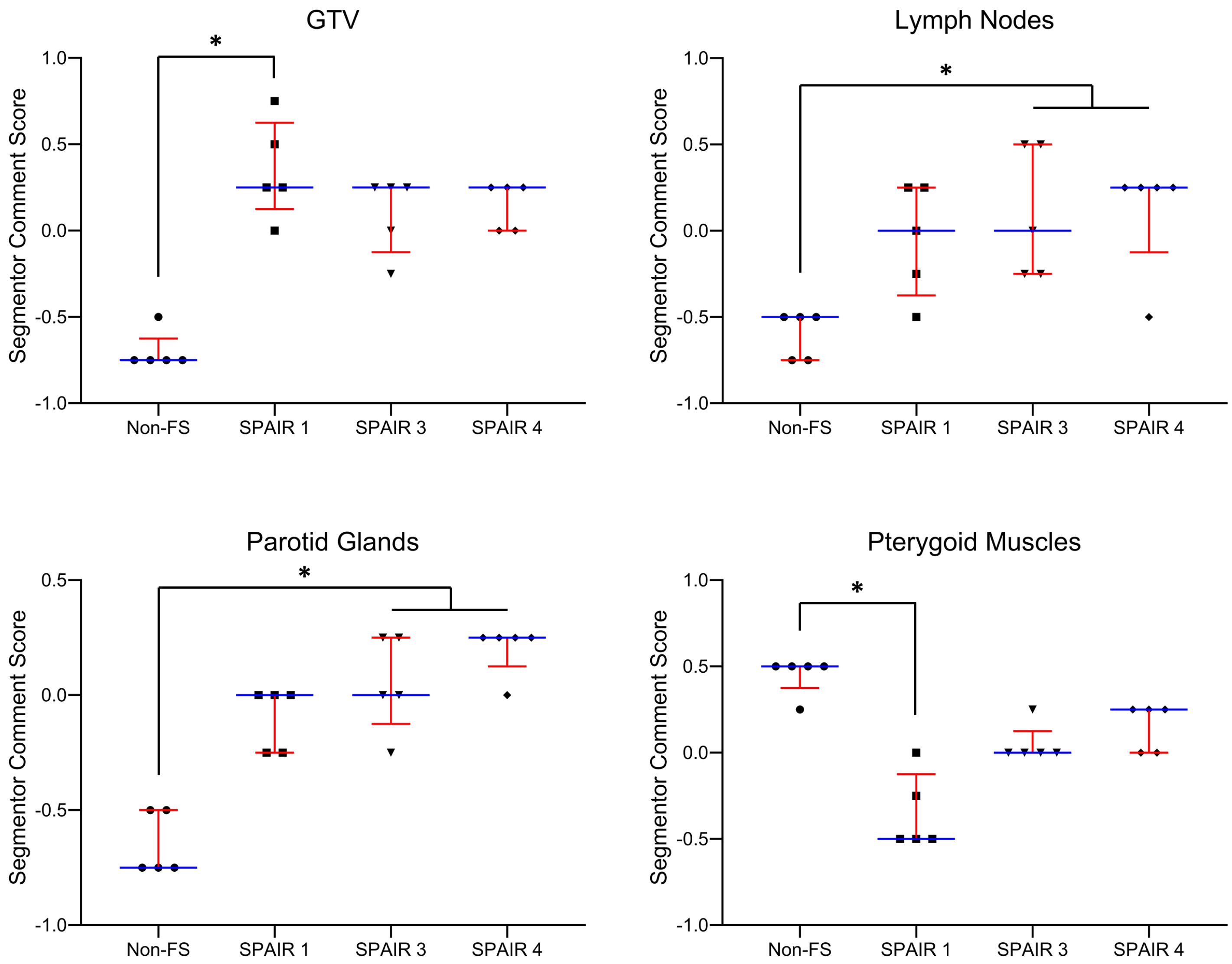

Figure S9: Segmentor comment score for the gross primary tumor volume (GTV), lymph nodes (LN), parotid glands (PAR), and pterygoid muscles (PTY) in the non-suppressed and spectral attenuated inversion recovery (SPAIR) sequences. These values represent the net positive comments for each segmentor as a percent of total segmentor comments. Each point represents a patient image. Median grades are depicted by horizontal blue lines, and interquartile ranges are depicted by vertical red lines. An asterisk* indicates a significant difference (P<.05).

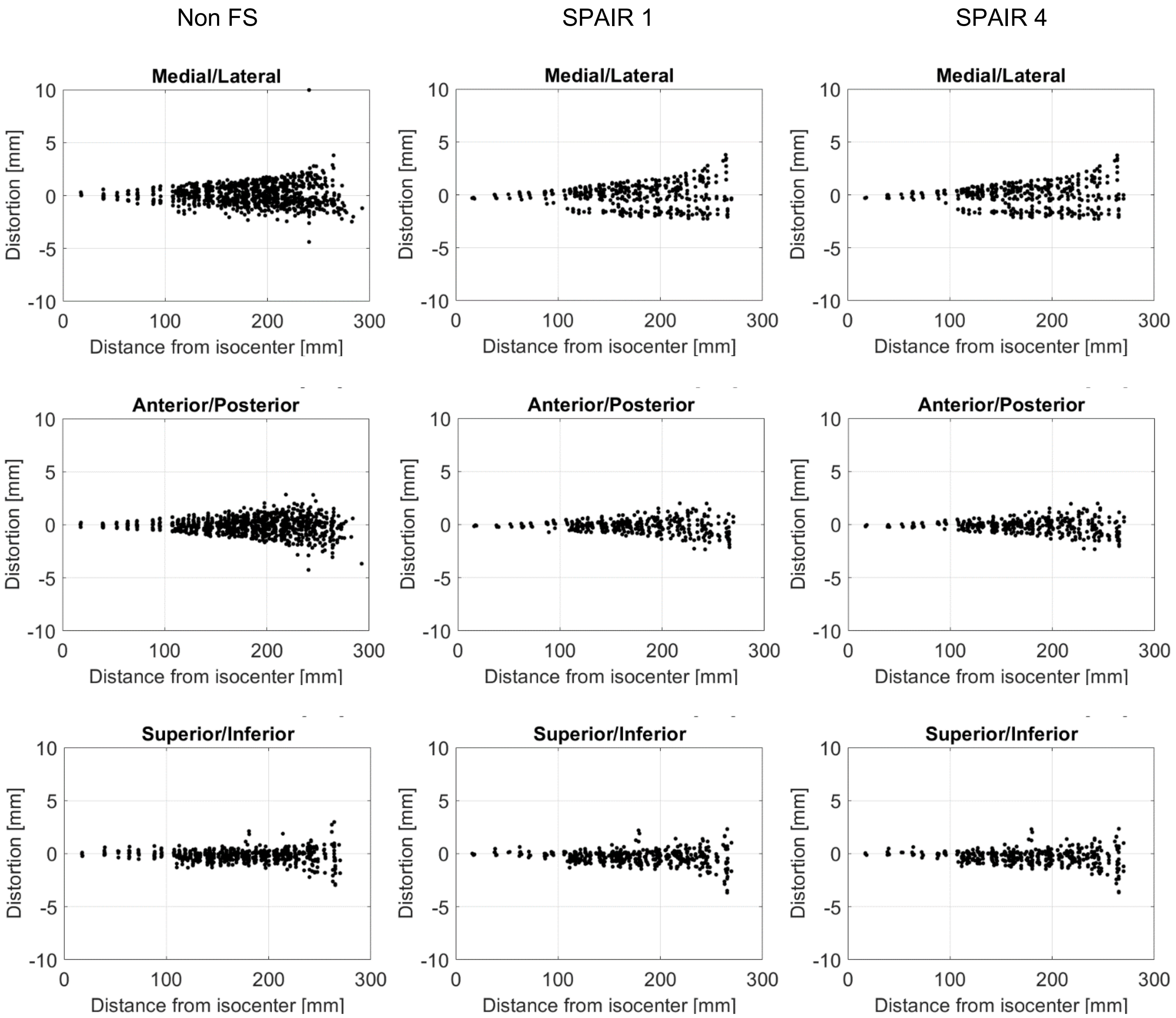

Figure S10: Geometric distortion distributions along the anatomic planes (rows) among the non-suppressed (Non-FS) and spectral attenuated inversion recovery (SPAIR) 1 and 4 sequences (columns). These data illustrate geometric distortion up to a diameter spherical volume of 500 mm, although the measurements in Table S6 are reflective of a diameter spherical volume of 300 mm, which is typically the maximum field of view used clinically. SPAIR 1 and 4 have very similar distributions, which is expected because of their very similar sequence parameters, but there are slight differences between the measurements, with a maximum discrepancy of 0.5 mm at 1 measurement location.

Appendix 1: Segmentor analysis

<u>Instructions for Contouring MRI Cases</u>

You will be assigned a Case Number, which you should use for each patient.

All cases are located in Raystation (Research).

At the bottom, you will see the individual MRI sequences which you can scroll through using the bar below. Double-click on the sequence that you want to work on. The first sequence is the non-fat-suppressed T2 image. The remaining images are the various iterations of fat-suppressed T2 images in random order. Double-clicking on one of the image viewers will maximize the window of the particular image plane. While you should use the Transverse plane to contour, you can refer to the other image planes to assist with visualization. When you are ready to contour, click the "New ROI geometry" icon on the header and select "Create new ROI". Name the contour "XXX_YY" where XXX is the name of the organ and YY are your initials. "Organ" can remain as the "Type" and choose the color of your liking.

The organs that should be contoured are the:

- Tumor GTV
- Involved or suspicious lymph nodes
- L and R parotid glands
- L and R Pterygoid muscles

As we want to ensure that each observer's contours are independent from each other, please do not look at other observer's contours. If you cannot locate a particular organ, it would be best to ask someone not involved with the study (refer to initial email for those participating). Additionally, this study will assess the ability of each sequence to visualize these organs. Thus, please refrain from looking at the patient's approved treatment plan. You may refer to any diagnostic exams and radiologist notes, as these are typically available to radiation oncologists during planning. Essentially, we would like to simulate the initial planning of a patient prescribed radiation. Time-permitting, please create each contour from scratch for each image, rather than propagating the contours between images of a patient.

Please see the next page for qualitative image assessment.

<u>Qualitative Image Assessment</u>

For each patient, rank each MR image according to preference for treatment planning (1 as lowest preference and 6 as highest preference). If you have the same preference for multiple images, give them the same rank. Provide the “Series description” which can be found by hovering your mouse over each image at the bottom of the screen.

MRN	MR1	MR2	MR3	MR4	MR5	MR6
	Description:<br><br>Rank:	Description:<br><br>Rank:	Description:<br><br>Rank:	Description:<br><br>Rank:	Description:<br><br>Rank:	Description:<br><br>Rank:
	Description:<br><br>Rank:	Description:<br><br>Rank:	Description:<br><br>Rank:	Description:<br><br>Rank:	Description:<br><br>Rank:	Description:<br><br>Rank:
	Description:<br><br>Rank:	Description:<br><br>Rank:	Description:<br><br>Rank:	Description:<br><br>Rank:	Description:<br><br>Rank:	Description:<br><br>Rank:
	Description:<br><br>Rank:	Description:<br><br>Rank:	Description:<br><br>Rank:	Description:<br><br>Rank:	Description:<br><br>Rank:	Description:<br><br>Rank:
	Description:<br><br>Rank:	Description:<br><br>Rank:	Description:<br><br>Rank:	Description:<br><br>Rank:	Description:<br><br>Rank:	Description:<br><br>Rank:

If you would like to elaborate on why an image was ranked high or low (such as presence of any artifacts or distortion), please list below. Provide the MRN and MR# with your finding.

Observer 1

1 = least preferred, 6 = most preferred

MRN	MR1	MR2	MR3	MR4	MR5	MR6
Pat1	Description: T2 3D tra<br><br>Rank: 5	Description: SPAIR1<br><br>Rank: 6	Description: SPAIR2<br><br>Rank: 3	Description: SPAIR3<br><br>Rank:1	Description: SPAIR4<br><br>Rank: 4	Description: SPAIR5<br><br>Rank: 2
Pat2	Description: T2 3D tra<br><br>Rank: 6	Description: SPAIR4<br><br>Rank: 2	Description: SPAIR2<br><br>Rank3	Description: SPAIR3<br><br>Rank:1	Description: SPAIR5<br><br>Rank: 5	Description: SPAIR1<br><br>Rank: 4
Pat3	Description: T2 3D tra<br><br>Rank: 2	Description: SPAIR1<br><br>Rank: 4	Description: SPAIR4<br><br>Rank: 5	Description: SPAIR2<br><br>Rank: 3	Description: SPAIR3<br><br>Rank: 6	Description: SPAIR5<br><br>Rank: 1
Pat4	Description: T2 3D tra<br><br>Rank: 1	Description: SPAIR1<br><br>Rank: 6	Description: SPAIR2<br><br>Rank: 2	Description: SPAIR4<br><br>Rank: 4	Description: SPAIR5<br><br>Rank: 3	Description: SPAIR3<br><br>Rank: 5
Pat5	Description: T2 3D tra<br><br>Rank: 1	Description: SPAIR1<br><br>Rank: 4	Description: SPAIR5<br><br>Rank: 6	Description: SPAIR4<br><br>Rank: 2	Description: SPAIR3<br><br>Rank: 5	Description: SPAIR2<br><br>Rank:3

Other comments:

Pat3 SPAIR1: impossible to see left pterygoid. Had to guess

Pat3 SPAIR5: couldn’t see the GTV at all

Pat4 T2 3D tra: good for normals, harder for GTV

Pat1 SPAIR2: really hard to see everything. Very fuzzy. Lymph node was impossible to see

Pat1 SPAIR5: difficult to make out left parotid

Pat2 SPAIR4: impossible to see GTV

Pat2 SPAIR2: very difficult to see GTV

Pat2 SPAIR3: very fuzzy

Pat2 SPAIR1: really hard to make out parotids

Pat5 SPAIR1: GTV and parotids great, very hard to see pterygoids

Observer 2

1 = least preferred, 6 = most preferred

MRN	MR1	MR2	MR3	MR4	MR5	MR6
Pat1	Description: T2 3D Tra<br><br>Rank: 5	Description: 3D T2 SPAIR1<br><br>Rank: 6	Description: 3D T2 SPAIR2<br><br>Rank: 5	Description: 3D T2 SPAIR3<br><br>Rank: 1	Description: 3D T2 SPAIR4<br><br>Rank: 5	Description: 3D T2 SPAIR5<br><br>Rank: 5
Pat2	Description: T2 3D Tra<br><br>Rank: 6	Description: 3D T2 SPAIR4<br><br>Rank: 2	Description: 3D T2 SPAIR2<br><br>Rank:3	Description: 3D T2 SPAIR3<br><br>Rank: 5	Description: 3D T2 SPAIR5<br><br>Rank:4	Description: 3D T2 SPAIR1<br><br>Rank:5
Pat3	Description: T2 3D Tra<br><br>Rank: 5	Description: 3D T2 SPAIR1<br><br>Rank: 2	Description: 3D T2 SPAIR4<br><br>Rank: 3	Description: 3D T2 SPAIR2<br><br>Rank: 3	Description: 3D T2 SPAIR3<br><br>Rank: 2	Description: 3D T2 SPAIR5<br><br>Rank: 3
Pat4	Description: T2 3D Tra<br><br>Rank: 6	Description: 3D T2 SPAIR1<br><br>Rank: 4	Description: 3D T2 SPAIR2<br><br>Rank: 3	Description: 3D T2 SPAIR4<br><br>Rank: 4	Description: 3D T2 SPAIR5<br><br>Rank: 5	Description: 3D T2 SPAIR3<br><br>Rank: 4
Pat5	Description: T2 3D Tra<br><br>Rank: 4	Description: 3D T2 SPAIR1<br><br>Rank: 3	Description: 3D T2 SPAIR5<br><br>Rank: 2	Description: 3D T2 SPAIR4<br><br>Rank: 4	Description: 3D T2 SPAIR3<br><br>Rank: 4	Description: 3D T2 SPAIR2<br><br>Rank: 5

Observer 3

1 = least preferred, 6 = most preferred

MRN	MR1	MR2	MR3	MR4	MR5	MR6
Pat1	Description: T2 3D Tra<br><br>GTV and LNs hard. Parotids very hard. Pterygoids good.<br><br>Rank: 1	Description: SPAIR1<br><br>GTV ok. LN hard. Parotids hard. Pterygoids hard.<br><br>Rank: 2	Description: SPAIR2<br><br>GTV ok. LN hard. Parotids ok. Pterygoids good.<br><br>Rank: 3	Description: SPAIR3<br><br>GTV ok. LN ok. Parotids good. Pterygoids ok.<br><br>Rank: 6	Description: SPAIR4<br><br>GTV ok. LN hard. Parotids good. Pterygoids ok.<br><br>Rank: 4	Description: SPAIR5<br><br>GTV ok. LN hard. Parotids good. Pterygoids good.<br><br>Rank: 5
Pat2	Description: T2 3D Tra<br><br>GTV hard. LNs hard. Parotids hard. Pterygoids good.<br><br>Rank: 1	Description: SPAIR4<br><br>GTV and LNs good. Parotids and pterygoids good.<br><br>Rank: 6	Description: SPAIR2<br><br>GTV and LNs good. Parotids and pterygoids fair.<br><br>Rank: 4	Description: SPAIR3<br><br>GTV and LNs fair. Parotids and pterygoids good.<br><br>Rank: 5	Description: SPAIR5<br><br>GTV and LNs fair. Parotids and pterygoids good.<br><br>Rank: 3	Description: SPAIR1<br><br>GTV fair. LNs hard. Parotids good. Pterygoids fair.<br><br>Rank: 2
Pat3	Description: T2 3D Tra<br><br>Pterygoids good. All other structures very hard.<br><br>Rank: 1	Description: SPAIR1<br><br>GTV and LN good. Pterygoids fair. Parotids hard.<br><br>Rank: 2	Description: SPAIR4<br><br>GTV and LN good. Pterygoids and parotids fair.<br><br>Rank: 5	Description: SPAIR2<br><br>GTV was fair. LN was great. Parotids and pterygoids were hard.<br><br>Rank: 3	Description: SPAIR3<br><br>GTV good. LN great. Parotids and pterygoids fair.<br><br>Rank: 6	Description: SPAIR5<br><br>GTV was fair. LN was good. Parotid and Pteryoids were fair.<br><br>Rank: 4
Pat4	Description: T2 3D Tra<br><br>Really hard to see GTV. All other structures hard.<br><br>Rank: 1	Description: SPAIR1<br><br>GTV fair. Parotids and pterygoids hard.<br><br>Rank: 2	Description: SPAIR2<br><br>Fair GTV. Parotids hard. Pterygoids really hard.<br><br>Rank: 3	Description: SPAIR4<br><br>All structures are good.<br><br>Rank: 4	Description: SPAIR5<br><br>Best sequence for all structures.<br><br>Rank: 6	Description: SPAIR3<br><br>GTV is the best. Pterygoids and parotids are good.<br><br>Rank: 5
Pat5	Description: T2 3D Tra<br><br>GTV and LN hard. Parotids and pterygoids good.<br><br>Rank: 1	Description: SPAIR1<br><br>GTV and LN good. Parotids and pterygoids hard.<br><br>Rank: 2	Description: SPAIR5<br><br>GTV and LN good. Parotids and pterygoids hard.<br><br>Rank: 4	Description: SPAIR4<br><br>GTV and LN good. Parotids and pterygoids the best.<br><br>Rank: 6	Description: SPAIR3<br><br>GTV and LN good. Parotids and pterygoids fair.<br><br>Rank: 3	Description: SPAIR2<br><br>GTV and LN good. Parotids great and pterygoids fair.<br><br>Rank: 5

Other comments:

Structures were hard to contour when the tissue around the structure had the same texture and contrast. Enhancement made the GTV and LNs easier. Pat1, Pat2: GTV and LNs were difficult in these cases. Pat3: artifact made the L pterygoid hard for this case.

Notes: (very) good = positive; (very) hard = negative; fair/okay = neutral

Observer 4

1 = least preferred, 6 = most preferred

MRN	MR1	MR2	MR3	MR4	MR5	MR6
Pat1	Description:<br>T2 3D Tra<br><br>Can't see parotid<br>Easier to see LN remnant<br>Had to imagine parts of GTV<br><br>Rank: 4	Description:<br>SPAIR1<br><br>Can't see LN remnant<br>Easiest to see GTV<br><br>Rank: 6	Description:<br>SPAIR2<br><br>Can't see LN remnant, had to hypothetically create<br>Second easiest to see GTV; easiest for parotid<br><br>Rank: 5	Description:<br>SPAIR3<br><br>Can't see LN remnant, had to hypothetically create<br><br>Rank: 3	Description:<br>SPAIR4<br><br>Can't see LN remnant, had to hypothetically create<br><br>Rank: 2	Description:<br>SPAIR5<br><br>Can't see LN remnant, had to hypothetically create<br>Had to imagine parts of GTV<br><br>Rank: 1
Pat2	Description:<br>T2 3D Tra<br><br>Had to guess parotid gland; had to imagine GTV<br><br>Rank: 1	Description:<br>SPAIR4<br><br>Fourth easiest to see GTV<br><br>Rank: 3	Description:<br>SPAIR2<br><br>Easiest to see parotid gland<br>Second easiest to see GTV<br><br>Rank: 5	Description:<br>SPAIR3<br><br>Third easiest to see GTV<br><br>Rank: 4	Description:<br>SPAIR5<br><br>Difficult to see GTV<br><br>Rank: 2	Description:<br>SPAIR1<br><br>Easiest to see GTV<br><br>Rank: 6
Pat3	Description:<br>T2 3D Tra<br><br>Impossible to see parotids and lymph nodes; had to guess to see them<br>hard to see GTV<br><br>Rank: 1	Description:<br>SPAIR1<br><br>Second easiest to see parotids<br>Easiest to see GTV<br><br>Rank: 6	Description:<br>SPAIR4<br><br>Easiest to see parotid gland<br>Second easiest to see GTV<br><br>Rank: 5	Description:<br>SPAIR2<br><br>Moderately easy to see GTV, but not as easily as base scan or 20201104<br><br>Rank: 4	Description:<br>SPAIR3<br><br>Reasonably good series<br><br>Rank: 3	Description:<br>SPAIR5<br><br>most challenging to see GTV<br><br>Rank: 2
Pat4	Description:<br>T2 3D Tra<br><br>Very hard to see parotid glands<br><br>Rank: 1	Description:<br>SPAIR1<br><br>Challenging to see pterygoids fully; easiest to see parotids<br>Third easiest to see GTV<br><br>Rank: 4	Description:<br>SPAIR2<br><br>Harder to see parotids than others<br>Easiest to see GTV<br><br>Rank: 6	Description:<br>SPAIR4<br><br>Very blurry<br>Third easiest to see GTV<br><br>Rank: 3	Description:<br>SPAIR5<br><br>Easiest to see parotid gland<br>Second easiest to see GTV<br><br>Rank: 5	Description:<br>SPAIR3<br><br>Challenging to see parotids<br><br>Rank: 2
Pat5	Description:<br>T2 3D Tra<br><br>Impossible to see parotids; had to guess to see them<br>Impossible to see LN<br><br>Rank: 1	Description:<br>SPAIR1<br><br>Easiest to see GTV<br><br>Rank: 6	Description:<br>SPAIR5<br><br>So blurry<br>Reasonably easy to see GTV<br><br>Rank: 2	Description:<br>SPAIR4<br><br>Very blurry<br>Third easiest to see GTV<br><br>Rank: 3	Description:<br>SPAIR3<br><br>Reasonably good series<br><br>Rank: 4	Description:<br>SPAIR2<br><br>Second easiest to see GTV<br><br>Rank: 5

Notes: 5th and 6th easiest = negative; 1st and 2nd easiest = positive; 3rd and 4th easiest = neutral

Observer 5

1 = least preferred, 6 = most preferred

MRN	MR1	MR2	MR3	MR4	MR5	MR6
Pat 1	Description: T2 3D Tra<br><br>Rank: 1	Description: 3D T2 SPAIR1<br><br>Rank: 6	Description: 3D T2 SPAIR2<br><br>Rank: 4	Description: 3D T2 SPAIR3<br><br>Rank: 5	Description: 3D T2 SPAIR4<br><br>Rank: 3	Description: 3D T2 SPAIR5<br><br>Rank: 2
Pat2	Description: T2 3D Tra<br><br>Rank: 1	Description: 3D T2 SPAIR4<br><br>Rank: 6	Description: 3D T2 SPAIR2<br><br>Rank: 5	Description: 3D T2 SPAIR3<br><br>Rank: 4	Description: 3D T2 SPAIR5<br><br>Rank: 3	Description: 3D T2 SPAIR1<br><br>Rank: 2
Pat3	Description: T2 3D Tra<br><br>Rank: 1	Description: 3D T2 SPAIR1<br><br>Rank: 6	Description: 3D T2 SPAIR4<br><br>Rank: 4	Description: 3D T2 SPAIR2<br><br>Rank: 3	Description: 3D T2 SPAIR3<br><br>Rank: 5	Description: 3D T2 SPAIR5<br><br>Rank: 2
Pat4	Description: T2 3D Tra<br><br>Rank: 1	Description: 3D T2 SPAIR1<br><br>Rank: 2	Description: 3D T2 SPAIR2<br><br>Rank: 4	Description: 3D T2 SPAIR4<br><br>Rank: 3	Description: 3D T2 SPAIR5<br><br>Rank: 5	Description: 3D T2 SPAIR3<br><br>Rank: 6
Pat5	Description: T23D Tra<br><br>Rank: 1	Description: 3D T2 SPAIR1<br><br>Rank: 6	Description: 3D T2 SPAIR5<br><br>Rank: 5	Description: 3D T2 SPAIR4<br><br>Rank: 4	Description: 3D T2 SPAIR3<br><br>Rank: 3	Description: 3D T2 SPAIR2<br><br>Rank: 2

Other comments:

For all T2 3D Tra: the muscles were well visualized but all other structures were significantly more difficult to evaluate.

Appendix 2: MR physicist analysis of SPAIR image quality

There are 5 SPAIR T2 images and 1 non-fat-suppressed T2 image for each patient. While looking through each image, please fill out the chart on the following pages with information about the image quality and presence of artifacts. Regarding the chart:

- List the series name/description (T2, SPAIR1, SPAIR2, SPAIR3, SPAIR4, SPAIR5)
- List the level of fat suppression (low, moderate, high)
- List any artifacts and provide the severity of the artifact (low, moderate, high)
- Provide any additional notes you may have about the image quality (i.e. clarity, qualitative contrast, etc.)
- For the SPAIR images (excluding the non-fat-suppressed T2), provide the relative rank of the overall image quality considering all artifacts and level of fat suppression
    - 1 for worst image quality, 5 for the best image quality

MR Physicist 1

MRN: Pat1			
Series Name:	Image Quality Assessment	Other Notes	Rank
T2 Tra (MRTC 6 min)	Fat Suppression: N/A<br><br>Artifacts (list and provide severity)<br>Minor inhomogeneity artifacts (hyperintensity/dropout) near dental metal and IV port on chest	Without fat sat, more difficult to determine gland boundaries & harder to distinguish gland from muscle based on signal intensity	
3D T2 SPAIR5	Fat Suppression: Modest, anterior burnout on lowest 70 slices of 250<br><br>Artifacts (list and provide severity); herringbone artifact and some Gibbs ringing near muscle and gland structures, 2 severity		2
3D T2 SPAIR1	Fat Suppression: Good posteriorly from roof of nasal cavity down to C4, good anteriorly from top slice to C5, then some burnout of anterior skin until C7, then no fat sat<br><br>Artifacts (list and provide severity) burnout (water saturation) anterior to mouth due to susceptibility artifact from positioning device	Easy to distinguish glands & other structures. Skin contour preserved for most of the image stack. Images appear a little smooth	3
3D T2 SPAIR2	Fat Suppression:<br>Good overall, mild burnout in anterior only for lowest 35 slices, signal loss near positioning device<br><br>Artifacts (list and provide severity): Herringbone artifact (fine lines) on both sides of jaw, severity 3, would interfere with auto segmentation. Ringing (Gibbs artifact) seen near muscle/fat interfaces, lowers apparent resolution, severity 2	Gland conspicuity is good, but ringing and blurriness make this more difficult to enjoy.	2
3D T2 SPAIR3	Fat Suppression: Good overall, mild burnout in anterior only for lowest 35 slices, mild signal loss near positioning device<br><br>Artifacts (list and provide severity): No obvious ringing or Gibbs artifact,	Good differentiation between gland, muscle and fat<br>Image sharpness looks good	5
3D T2 SPAIR4	Fat Suppression: Very good. mild burnout in anterior only for lowest 35 slices, mild signal loss near positioning device<br><br>Artifacts (list and provide severity): No obvious ones except signal loss	Good differentiation between gland, muscle and fat<br>Image sharpness looks good, maybe not quite as good as SPAIR3	4

MRN: Pat2			
Series Name:	Image Quality Assessment	Other Notes	Rank
T2 Tra	Fat Suppression: N/A<br><br>Artifacts (list and provide severity): signal dropouts near immobilization device	SNR high, conspicuity of glands and tumor lower<br>Apparent metal artifact or inhomogeneity near right shoulder causing artifacts in all scans	
3D T2 SPAIR4	Fat Suppression: Inhomogeneous, burnout posteriorly from slices 80-130, no fat sat posteriorly below slice 80 or above slice 150<br><br>Artifacts (list and provide severity): Bright vessels and some blurriness of glands/tumor, severity 3 (moderate).	Glands visible but bright vascular structures nearby may limit segmentation	5
3D T2 SPAIR2	Fat Suppression: Signal burnout anterior up to slice 80 and posterior slices 100-150<br><br>Artifacts (list and provide severity): flame artifact (zebra pattern) slices 120-140 posterior right side, severity 2 because not near tumor but would be higher if covering anatomy of interest; herringbone artifact near jaw, severity low (1)	Some blurriness in tumor & gland depiction	2
3D T2 SPAIR3	Fat Suppression: Aggressive<br>Anterior burnout to slice 80, lateral posterior burnout (loss of skin contour) slices 110-145<br><br>Artifacts (list and provide severity):	Gland and tumor conspicuity are good	3
3D T2 SPAIR5	Fat Suppression:<br>Anterior burnout to slice 100, posterolateral burnout slices 120-150<br><br>Artifacts (list and provide severity): herringbone on both sides of jaw, severity 2, medium, some Gibbs ringing near structures.	Some blurriness in gland depiction	1
3D T2 SPAIR1	Fat Suppression:<br>Anterior burnout to slice 90 including trachea from slices 50-80<br>Posterolateral burnout slices 110-145<br><br>Artifacts (list and provide severity):	Bright vascular structures obscure some boundaries of glands & tumor	3

MRN: Pat3			
Series Name:	Image Quality Assessment	Other Notes	Rank
3D T2	Fat Suppression: N/A<br><br>Artifacts (list and provide severity): significant signal dropout due to dental metal	Tumor conspicuity is low	
3D T2 SPAIR1	Fat Suppression:<br>Anterior burnout from slice 30-80<br><br>Artifacts (list and provide severity): loss of fat sat near dental metal	Gland conspicuity is still good even where fat sat is failing due to metal	4
3D T2 SPAIR4	Fat Suppression: Very inhomogeneous. Burnout anteriorly from slice 30-100, also signal dropoff at lateral shoulders at bottom of imaging stack<br><br>Artifacts (list and provide severity): loss of fat sat and signal near dental metal, obscuring anatomy, high severity (3? 5?)	Gland boundary almost obscured by fat sat artifacts	2
3D T2 SPAIR2	Fat Suppression:<br>Anterior burnout from slice 20-90<br><br>Artifacts (list and provide severity):<br>flame artifact (zebra pattern) slices 110-140 posterior right side, severity 2 because not near tumor but would be higher if covering anatomy of interest; loss of fat sat and signal near dental metal, obscuring anatomy, high severity (3? 5?)	Gland boundary almost obscured by fat sat artifacts	1
3D T2 SPAIR3	Fat Suppression:<br>Anterior burnout from slice 30-80<br><br>Artifacts (list and provide severity): loss of fat sat and signal near dental metal, obscuring anatomy, high severity (3? 5?)	Gland boundary almost obscured by fat sat artifacts	5
3D T2 SPAIR5	Fat Suppression: Inhomogenous; Anterior burnout slices 60-<br>100<br><br>Artifacts (list and provide severity): Respiratory artifact due to lower placement of imaging stack obscures chest wall from slices 1-60 ; herringbone artifact on anterior cheeks slices 120-180 low severity.	Gland boundary obscured by fat sat failure	2

MRN: Pat4			
Series Name:	Image Quality Assessment	Other Notes	Rank
T2 3D Tra	Fat Suppression:N/A<br><br>Artifacts (list and provide severity)	Gland and tumor conspicuity are low	
3D T2 SPAIR1	Fat Suppression:<br>Burnout posteriorly slices 180-130 then losing fat sat posteriorly below slice 130<br><br>Artifacts (list and provide severity):	Good parotid gland conspicuity, lower contrast for some other structures near muscle. (Image looks a little flat.)	4
3D T2 SPAIR2	Fat Suppression:<br>Posterior burnout from slice 130-190, no posterior fat sat below slice 130<br><br>Artifacts (list and provide severity): flame artifact posteriorly slices 147-114 severity 2.; herringbone/ringing artifact slices 180-130 obscuring gland interfaces, severity high (3)	Gland edges would be disrupted by artifact	1
3D T2 SPAIR4	Fat Suppression:<br>Anterior burnout slice 5-75, posterior burnout slices 84-120<br><br>Artifacts (list and provide severity)	Gland conspicuity good, no apparent artifacts; even a nice view of the optic nerves with fat sat near slice 200	5
3D T2 SPAIR5	Fat Suppression:<br>Posterior burnout slice 150-205<br>Anterior burnout or indistinct slices 85-145<br><br>Artifacts (list and provide severity): herringbone artifacts slices 150-180 severity 1 but adjacent to glands	Good aortic arch depiction (irrelevant but nice)	2
3D T2 SPAIR3	Fat Suppression:<br>Anterior burnout slices 60-130<br>Posterior burnout from 200 down to 135 where fat sat fails<br><br>Artifacts (list and provide severity)	Mostly dark vessels help visualization	3

MRN: Pat5			
Series Name:	Image Quality Assessment	Other Notes	Rank
T2 3D Tra	Fat Suppression: N/A<br><br>Artifacts (list and provide severity)	Minor artifact from dental metal	
3D T2 SPAIR1	Fat Suppression:<br>Anterior burnout slices 20-75<br>Posterior burnout from slices 100-140<br><br>Artifacts (list and provide severity)	A little blurry	2
3D SPAIR5	Fat Suppression:<br>Anterior burnout slice 60-95, not as severe as usual<br><br>Artifacts (list and provide severity): herringbone on cheeks and edges of glands severity 2.	Mild breakdown of fat sat near dental metal, not much anatomy obscured	2
3D T2 SPAIR4	Fat Suppression:<br>Anterior burnout slices 5-65<br>; posterior burnout slices 85-120<br><br>Artifacts (list and provide severity)	Good gland & tumor conspicuity	5
3D T2 SPAIR3	Fat Suppression:<br>Anterior burnout or missing contour from slice 1-55; Posterior burnout from slices 55-95<br><br>Artifacts (list and provide severity): low SNR posterior neck region		3
3D T2 SPAIR2	Fat Suppresion:<br>Posterior burnout slices 55-100; anterior burnout slices 5-50 (bad burnout on slices 10-20)<br><br>Artifacts (list and provide severity):<br>Flame artifact posterior slices 65-85	CNR very good (high conspicuity)	3

MR Physicist 2

MRN: Pat1			
Series Name:	Image Quality Assessment	Other Notes	Rank
SPAIR1	Fat Suppression: moderate<br><br>Artifacts (list and provide severity): low	All comments for HN area only, not off isocenter.	4
SPAIR4	Fat Suppression: high<br><br>Artifacts (list and provide severity): low	Higher T2 contrast	5
SPAIR2	Fat Suppression: moderate<br><br>Artifacts (list and provide severity):<br>filtering - moderate	*filtering – edge effects likely cause by echo sampling scheme.	2
SPAIR3	Fat Suppression: moderate<br><br>Artifacts (list and provide severity): low		3
SPAIR5	Fat Suppression: moderate<br><br>Artifacts (list and provide severity):<br>filtering – moderate<br>partial volume – moderate<br>Due to noticeably thicker slices		1

MRN: Pat2			
Series Name:	Image Quality Assessment	Other Notes	Rank
SPAIR1	Fat Suppression: moderate<br><br>Artifacts (list and provide severity): low	All comments for HN area only, not off isocenter.	4
SPAIR4	Fat Suppression: high<br><br>Artifacts (list and provide severity): low	Higher T2 contrast	5
SPAIR2	Fat Suppression: moderate<br><br>Artifacts (list and provide severity):<br>filtering – moderate	*filtering – edge effects likely cause by echo sampling scheme.	2
SPAIR3	Fat Suppression: moderate<br><br>Artifacts (list and provide severity): low		3
SPAIR5	Fat Suppression: moderate<br><br>Artifacts (list and provide severity):<br>filtering – moderate<br>partial volume - moderate		1

MRN: Pat3			
Series Name:	Image Quality Assessment	Other Notes	Rank
SPAIR1	Fat Suppression: moderate<br><br>Artifacts (list and provide severity):<br>Metal - moderate	All comments for HN area only, not off isocenter.<br><br>Failure around metal similar for all images	4
SPAIR4	Fat Suppression: high<br><br>Artifacts (list and provide severity):<br>Metal - moderate	Higher T2 contrast	5
SPAIR2	Fat Suppression: moderate<br><br>Artifacts (list and provide severity):<br>Metal - moderate<br>filtering – moderate<br>aliased phase - high	*filtering – edge effects likely cause by echo sampling scheme.	1
SPAIR3	Fat Suppression: moderate<br><br>Artifacts (list and provide severity):<br>Metal - moderate		3
SPAIR5	Fat Suppression: moderate<br><br>Artifacts (list and provide severity):<br>Metal - moderate<br>filtering – moderate<br>partial volume - moderate		2

MRN: Pat4			
Series Name:	Image Quality Assessment	Other Notes	Rank
SPAIR1	Fat Suppression: moderate<br><br>Artifacts (list and provide severity): low	All comments for HN area only, not off isocenter.	4
SPAIR4	Fat Suppression: high<br><br>Artifacts (list and provide severity): low	Higher T2 contrast	5
SPAIR2	Fat Suppression: moderate<br><br>Artifacts (list and provide severity):<br>filtering – moderate	*filtering – edge effects likely cause by echo sampling scheme.	2
SPAIR3	Fat Suppression: moderate<br><br>Artifacts (list and provide severity): low		3
SPAIR5	Fat Suppression: moderate<br><br>Artifacts (list and provide severity):<br>filtering – moderate<br>partial volume - moderate		1

MRN: Pat5			
Series Name:	Image Quality Assessment	Other Notes	Rank
SPAIR1	Fat Suppression: low<br><br>Artifacts (list and provide severity): low	All comments for HN area only, not off isocenter.	3
SPAIR4	Fat Suppression: high<br><br>Artifacts (list and provide severity): low	Higher T2 contrast	5
SPAIR2	Fat Suppression: moderate<br><br>Artifacts (list and provide severity):<br>filtering – moderate<br>aliased phase - moderate	*filtering – edge effects likely cause by echo sampling scheme.	2
SPAIR3	Fat Suppression: moderate<br><br>Artifacts (list and provide severity): low		4
SPAIR5	Fat Suppression: moderate<br><br>Artifacts (list and provide severity):<br>filtering – moderate<br>partial volume - moderate		1

Appendix 3: Rubric for overall sequence scoring

For each metric that was analyzed, a score was determined for each sequence-structure pair. Each sequence-structure pair received a score between 1 and 4, where 4 corresponded to the pair with the best performance. Sequence-structure pairs could only receive a higher score than the other pairs if the difference was statistically significant ($p<0.05$) compared to all other sequence-structure pairs with a lower score. For example, if the SNR of Sequence A was statistically higher than B and C, then sequence A would be scored higher than B and C. However, if Sequence A was statistically higher than B but not C, and Sequence C was not statistically higher than B, then all 3 sequences would receive the same score. Sequence-structure pairs that received the same score were rescaled to the average rank between them. For example, if a scoring distribution was scored as 4,4,4,1 it was rescaled to 3,3,3,1, where 3=(4+3+2)/3. For clarity, these will be regarded as normalized metric scores

For each analysis category (SNR and CNR, conspicuity, etc.), the normalized metric scores within that category were summed for each sequence-structure pair to produce category scores. The category score was then renormalized between 1 and 4 (4 corresponding to highest category score), according to its rank relative to the same structure among the other sequences. For clarity, these will be regarded as normalized category scores. This normalization was performed so that a category with more metrics (such as SNR and CNR measurements) would be weighed the same in the overall analysis as a category with fewer metrics (such as conspicuity).

The normalized category scores for each sequence-structure pair were then summed and normalized to determine the total score and normalized total score. These scores were used to compare the overall image quality for each structure among the sequences. Additionally, the total score for each structure within a sequence was summed and normalized to determine the combined total score and combined normalized score. These scores were used to compare the overall image quality across structures among the sequences. Refer to Figure 1 in the manuscript for a graphical depiction of the scoring.
